# Piezo2 voltage-block regulates mechanical pain sensitivity

**DOI:** 10.1093/brain/awae227

**Published:** 2024-07-10

**Authors:** Oscar Sánchez-Carranza, Sampurna Chakrabarti, Johannes Kühnemund, Fred Schwaller, Valérie Bégay, Jonathan Alexis García-Contreras, Lin Wang, Gary R Lewin

**Affiliations:** Molecular Physiology of Somatic Sensation Laboratory, Max Delbrück Center for Molecular Medicine in the Helmholtz Association (MDC), Berlin 10409, Germany; Molecular Physiology of Somatic Sensation Laboratory, Max Delbrück Center for Molecular Medicine in the Helmholtz Association (MDC), Berlin 10409, Germany; Molecular Physiology of Somatic Sensation Laboratory, Max Delbrück Center for Molecular Medicine in the Helmholtz Association (MDC), Berlin 10409, Germany; Molecular Physiology of Somatic Sensation Laboratory, Max Delbrück Center for Molecular Medicine in the Helmholtz Association (MDC), Berlin 10409, Germany; Molecular Physiology of Somatic Sensation Laboratory, Max Delbrück Center for Molecular Medicine in the Helmholtz Association (MDC), Berlin 10409, Germany; Molecular Physiology of Somatic Sensation Laboratory, Max Delbrück Center for Molecular Medicine in the Helmholtz Association (MDC), Berlin 10409, Germany; Molecular Physiology of Somatic Sensation Laboratory, Max Delbrück Center for Molecular Medicine in the Helmholtz Association (MDC), Berlin 10409, Germany; Molecular Physiology of Somatic Sensation Laboratory, Max Delbrück Center for Molecular Medicine in the Helmholtz Association (MDC), Berlin 10409, Germany; Charité-Universitätsmedizin Berlin, 10117 Berlin, Germany; German Center for Mental Health (DZPG), partner site Berlin, 10117 Berlin, Germany

**Keywords:** ion channels, mechanotransduction, human developmental disorders, pain, voltage-block

## Abstract

PIEZO2 is a trimeric mechanically-gated ion channel expressed by most sensory neurons in the dorsal root ganglia. Mechanosensitive PIEZO2 channels are also genetically required for normal touch sensation in both mice and humans. We previously showed that PIEZO2 channels are also strongly modulated by membrane voltage. Specifically, it is only at very positive voltages that all channels are available for opening by mechanical force. Conversely, most PIEZO2 channels are blocked at normal negative resting membrane potentials. The physiological function of this unusual biophysical property of PIEZO2 channels, however, remained unknown.

We characterized the biophysical properties of three PIEZO2 ion channel mutations at an evolutionarily conserved arginine (R2756). Using genome engineering in mice we generated *Piezo2*^R2756H/R2756H^ and *Piezo2*^R2756K/R2756K^ knock-in mice to characterize the physiological consequences of altering PIEZO2 voltage sensitivity *in vivo*. We measured endogenous mechanosensitive currents in sensory neurons isolated from the dorsal root ganglia and characterized mechanoreceptor and nociceptor function using electrophysiology. Mice were also assessed behaviourally and morphologically.

Mutations at the conserved Arginine (R2756) dramatically changed the biophysical properties of the channel relieving voltage block and lowering mechanical thresholds for channel activation. *Piezo2^R2756H/R2756H^* and *Piezo2^R2756K/R2756K^* knock-in mice that were homozygous for gain-of-function mutations were viable and were tested for sensory changes. Surprisingly, mechanosensitive currents in nociceptors, neurons that detect noxious mechanical stimuli, were substantially sensitized in *Piezo2* knock-in mice, but mechanosensitive currents in most mechanoreceptors that underlie touch sensation were only mildly affected by the same mutations. Single-unit electrophysiological recordings from sensory neurons innervating the glabrous skin revealed that rapidly-adapting mechanoreceptors that innervate Meissner's corpuscles exhibited slightly decreased mechanical thresholds in *Piezo2* knock-in mice. Consistent with measurements of mechanically activated currents in isolated sensory neurons essentially all cutaneous nociceptors, both fast conducting Aδ-mechanonociceptors and unmyelinated C-fibre nociceptors were substantially more sensitive to mechanical stimuli and indeed acquired receptor properties similar to ultrasensitive touch receptors in *Piezo2* knock-in mice. Mechanical stimuli also induced enhanced ongoing activity in cutaneous nociceptors in *Piezo2* knock-in mice and hyper-sensitive PIEZO2 channels were sufficient alone to drive ongoing activity, even in isolated nociceptive neurons. Consistently, *Piezo2* knock-in mice showed substantial behavioural hypersensitivity to noxious mechanical stimuli.

Our data indicate that ongoing activity and sensitization of nociceptors, phenomena commonly found in human chronic pain syndromes, can be driven by relieving the voltage-block of PIEZO2 ion channels. Indeed, membrane depolarization caused by multiple noxious stimuli may sensitize nociceptors by relieving voltage-block of PIEZO2 channels.

## Teaser

The PIEZO2 ion channel transduces mechanical stimuli into neural signals for touch and pain. Sánchez *et al.* show in mice that making PIEZO2 highly sensitive to mechanical stimuli renders pain receptors super-sensitive. This mechanism could underlie pathological mechanical hypersensitivity after injury.

**See Fernández-Trillo *et al.* (https://doi.org/10.1093/brain/awae290) for a scientific commentary on this article.**

## Introduction

**Figure 1:**
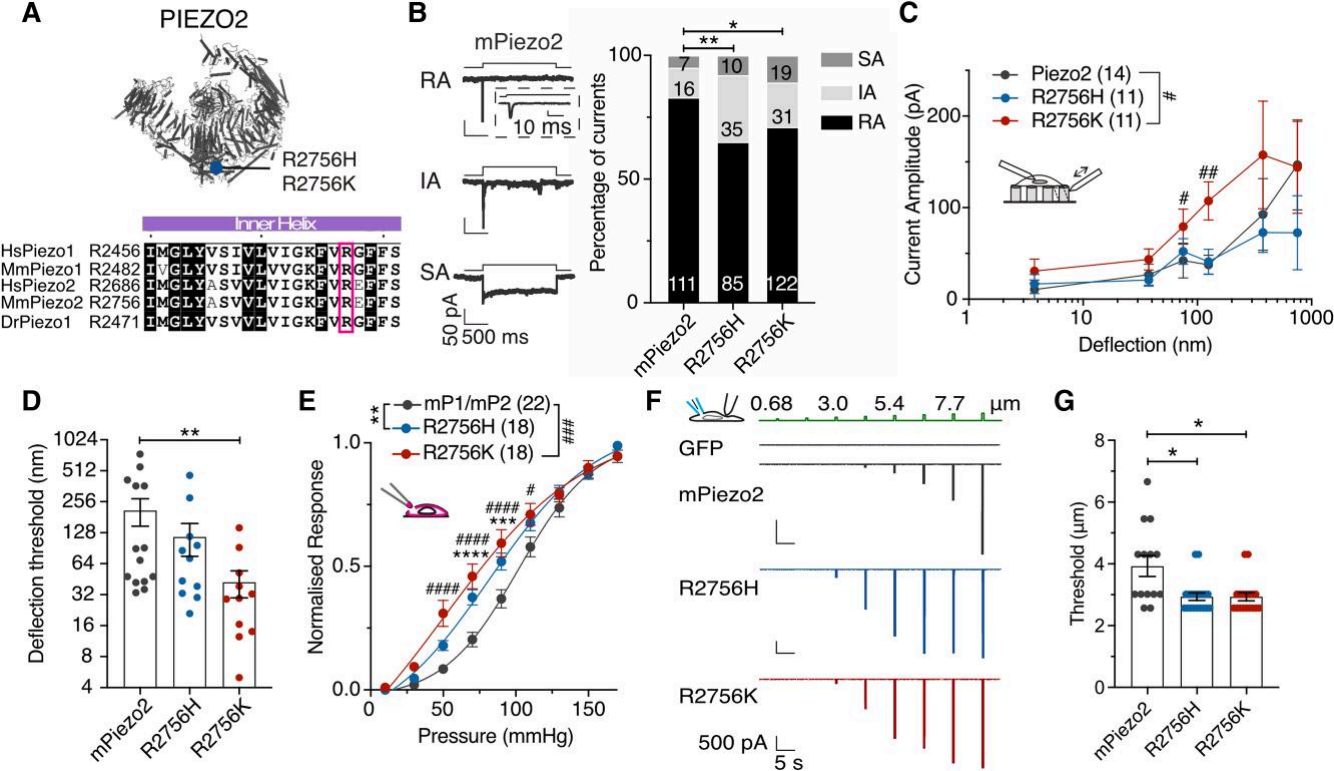
**Mutations in the R2756 of *mPiezo2* showed enhanced sensitivity to mechanical stimuli.** (**A**) *Top*: Structural model of PIEZO2 (PBD ID: 6KG7^13^) indicating the position of the R2756 (blue dot). *Bottom*: Residues alignment (using ESPript 3.0^14^) showing that the arginine (pink square) is conserved in PIEZO channels. (**B**) *Left*: Example traces of rapidly adapting (RA), intermediate adapting (IA) and slowly adapting (SA) currents from N2a*^Piezo1−/−^* cells overexpressing mPiezo2. *Right*: Proportion of RA currents is decreased in cells expressing mPiezo2 variants. Numbers represent the currents recorded (χ^2^ test; *P = 0.04, **P = 0.004). (**C**) Deflection-response relationships showing that R2756K mutant is more sensitive to mechanical stimuli compared to wild-type (Mann–Whitney test; ^#^*P* = 0.04, ^##^*P* = 0.008; two-way ANOVA indicated differences between variants; ^#^*P* = 0.04). (**D**) Deflection threshold was lower in the R2756K mutant (Kruskal–Wallis test; **P = 0.006). (**E**) Stretch-response curves of the chimeric channel variants. Peak currents were normalized according to their maximum (two-way ANOVA; **P = 0.003, ^###^*P* = 0.0002; with Sídák *post hoc* test; ***P = 0.0006, ****P < 0.0001, ^#^*P* = 0.03, ^####^*P* < 0.0001). (**F**) Representative traces of mechanosensitive currents from Piezo2 wild-type and mutants overexpressed in N2a*^Piezo1−/−^* cells evoked with the indentation method. (**G**) Mechanical threshold was reduced in Piezo2 mutants compared to control (Kruskal–Wallis test; *P < 0.05).

*Piezo2* is genetically required for normal touch sensation,^1-4^ and it is widely assumed that PIEZO2 channels form the conduction pore of native mechanosensitive currents that underlie touch receptor mechanosensitivity. However, deletion of *Piezo2* does not lead to complete loss of mechanosensitivity in all mechanoreceptors.^2,5-7^ This data is also consistent with the existence of other mechanically activated channels in mechanoreceptors, like ELKIN1, that may play a non-redundant role in touch.^8^ Importantly, the mechanosensitivity of almost all nociceptors is largely preserved in the absence of *Piezo2.*^5^ Work in nematodes has shown how genetic deletion of candidate mechanotransduction channels does not always provide definitive evidence that the protein forms the pore of the native mechanosensitive current.^9-11^ A powerful way to directly assess the participation of a channel in transduction is to change the biophysical properties of the endogenous channel with the prediction that native mechanosensitive currents should acquire these new biophysical properties.^10^ PIEZO channels are not only gated by mechanical stimuli, but are also controlled by membrane voltage. Thus, at physiological membrane potentials >90% of PIEZO channels cannot be opened by mechanical stimuli, but are made available at depolarized membrane potentials.^12^ We previously identified a single highly conserved arginine residue in PIEZO1 channels (mR2482) that when mutated effectively eliminates most of the PIEZO1 voltage-block^12^ (Fig. 1A). Interestingly, mutations in the same conserved residue of PIEZO2 (mPIEZO2; R2756; hPIEZO2 R2686) are associated with distal arthrogryposis, Gordon syndrome and the Marden-Walker syndrome, all of which are human developmental disorders.^15,16^ Here we show that each of these single mutations can abrogate the voltage-block of the PIEZO2 channel, dramatically increasing channel availability at physiological membrane potentials. We used mouse genetics to mutate the same site in the channel *in vivo* to investigate the effects of changing the channel properties on native mechanosensitive currents and their effects on sensory physiology. Surprisingly, we observed only minor effects on touch receptors, but the properties of mechanosensitive currents in nociceptors were dramatically sensitized in a way that reflected the changes in PIEZO2 channel function. Our data show how the voltage block of PIEZO2 serves to keep the mechanical threshold of nociceptors high so that they detect noxious and not innocuous mechanical stimuli. Furthermore, our data suggest a simple model whereby different kinds of noxious or sensitizing stimuli may drive nociceptor sensitization by releasing PIEZO2 channels from voltage-block.

## Materials and methods

### Animals

All experiments with mice were done in accordance with protocols reviewed and approved by the German Federal authorities (State of Berlin).

### Molecular biology

DNA constructs containing mPiezo2, mP1/mP2 (chimeric channel containing residues 1–2188 of mPiezo1 and 2472–2822 of mPiezo2)^12^ and the variants were purified from transformed bacteria grown in large-scale bacterial culture (50 ml Midiprep, PureYield™ Plasmid Midiprep System, Promega). The midipreps were made according to the manufacture's protocol. DNA quantification was measured using a NanoDrop 2000 (Thermofisher Scientific).

Insertion of point mutations in mPiezo2 and the chimeric channel mP1/mP2 were carried out using the Q5^®^ Site-Directed Mutagenesis Kit (NEB Inc.) according to the manufacture's indications. Specific primers for each mutant were used at 0.5 µM. Variant R2746H was generated using forward primer 5′-GAAATTTGTTCATGAGTTCTTCAG-3′, R2756C using forward primer 5′-GAAATTTGTTTGTGAGTTCTTCAG-3′ and R2756K using forward primer 5′-GAAATTTGTTAAAGAGTTCTTCAGTGGG-3′. For all mutants the same reverse primer was used, 5′-CCAATTACAAGGACAACAG-3′. PCR products were used as template for bacteria transformation and ampicillin resistant colonies were chosen and grown in large-scale bacterial culture for DNA purification. DNA plasmids were sequenced to verify the insertion of point mutations.

### Dorsal root ganglia culture

Dorsal root ganglia (DRG) neurons were collected from all the spinal segments in plating medium on ice [DMEM-F12 (Invitrogen) supplemented with L-glutamine (2 μM, Sigma-Aldrich), glucose (8 mg/ml, Sigma Aldrich), penicillin (200 U/ml)-streptomycin (200 μg/ml) and 10% fetal horse serum]. The DRGs were treated with collagenase IV (1 mg/ml, Sigma-Aldrich) for 1 h at 37°C and then washed three times with Ca^2+^- and Mg^2+^-free PBS. The samples were incubated with trypsin (0.05%, Invitrogen) for 15 min, at 37°C. After the enzymatic treatment, the collected tissue was triturated with a pipette tip and plated in a droplet of plating medium on the elastomeric pillar arrays precoated with laminin (4 μg/cm^2^, Invitrogen) as described by Poole *et al*.^17^ for the pillar arrays experiments (see the ‘Preparation of pillar arrays’ section). Cells were cultured overnight, and the electrophysiology experiments were preformed after 18–24 h of the dissection.

### Preparation of pillar arrays

Pillar arrays were prepared as previously described.^17-19^ Briefly, silanized negative masters were used as templates. Negative masters were covered with polydimethylsilozane (PDMS, SYLGARD^TM^ 184 silicone elastomer kit, Dow Corning Corporation) mixed with a curing agent at 10:1 ratio (elastomeric base:curing agent) and incubated for 30 min. Glass coverslips were placed on the top of the negative masters containing PDMS and baked for 1 h at 110°C. Pillar arrays were carefully peeled from the negative masters. The resulting radius- and length-size of individual pilus within the array was 1.79 µm and 5.8 µm, respectively. The elasticity and the spring constant of each pilus was 2.1 MPa and 251 pN-nm, respectively, as previously reported.^17-19^ Before use for cell culture, pillar arrays were plasma cleaned with a Femto low-pressure plasma system (Deiner Electronic) and coated with EHS laminin (20 µg/ml).

### Electrophysiology

Whole-cell patch clamp experiments were made from DRG neurons and transiently transfected N2a*^Piezo1−/−^* cells using pulled and heat-polished borosilicate glass pipettes (Harvard apparatus, 1.17 mm × 0.87 mm) with a resistance of 3–6 MΩ. All experiments were carried out at room temperature. The pipettes were pulled using a DMZ puller and filled with a solution containing (in mM): 110 KCl, 10 NaCl, 1 MgCl_2_, 1 EGTA and 10 HEPES. For recordings in DRG neurons QX-314 (Alomone Labs) at 1 μM was added. The pH was adjusted to 7.3 with KOH. The extracellular solution contained (in mM): 140 NaCl, 4 KCl, 2 CaCl_2_, 1 MgCl_2_, 4 glucose and 10 HEPES. The pH was adjusted to 7.4 with NaOH. Pipette and membrane capacitance were compensated using the auto-function of Patchmaster (HEKA, Elektornik) and series resistance was compensated to minimize voltage errors. Currents were evoked by mechanical stimuli at a holding potential of −60 mV; for details see the Supplementary material.

Current-clamp experiments were performed to classify sensory neurons into mechanoreceptors and nociceptors. Spontaneous activity was determined by recording the membrane potential of neurons for 20 s in the absence of current injection and mechanical stimulation. Cells firing action potentials in the absence of current injection were considered as responsive cells. For soma indentation assays using current-clamp, the mechanical threshold was defined as the minimum indentation stimulus that resulted in the first action potential. Neurons that did not fire action potentials were considered as non-responsive cells. For the resting membrane fluctuations, current clamp recordings were performed and the change in membrane potential (ΔEm) was calculated (maximum membrane potential peak − minimum membrane potential peak).

Currents and the biophysical parameters were analysed using FitMaster (HEKA, Elektornik).

### *Ex vivo* skin nerve

Cutaneous sensory fibre recordings were performed using the *ex vivo* skin nerve preparation. Mice were euthanized by CO_2_ inhalation for 2–4 min followed by cervical dislocation. We used the recently described tibial nerve preparation to record from single-units innervating the glabrous hindpaw skin.^20,21^ Details of recording methods and stimulation protocols can be found in the Supplementary material.

### Generation of *Piezo2^R2756H^* and *Piezo2^R2756K^* mice

Constitutive knock-in mice were generated using CRISPR-Cas9 technology by the ingenious targeting laboratory. For each mutant, gRNAs (guide RNAs) and ssDNA (single-stranded DNA) donors were designed. For mutant *Piezo2^R2756H^* was generated using the gRNA 5′-TGGAAGCTCTTCAAACATGATGG-3′ and the ssDNA donor 5′-TGCTGTCTCTTTCAGTATCATGGGATTGTATGCATCTGTTGTCCTTGTAATTGGGAAATTTGTTCATGAGTTCTCAGTGGGATCTCTCATTCCATCATGTTTGAAGAGCTTCCAAATGTGGACAGAATCTTGAAGTTGTGCACAGATATATTCCTCGTGAGGGAGACA-3′. Mice *Piezo2^R2756K^* was generated using the gRNA 5′-TTGTTCGTGAGTTCTTCAGTGGG-3′ and the ssDNA donor 5′-ACCATCTTCATCATTTTCTCCTTGCTGTCTCTTTCAGTATCATGGGATTGTATGCATCTGTTGTCCTTGTAATTGGGAAATTTGTTAAGGAGTTCTCAGTGGGATCTCTCATTCCATCATGTTTGAAGAGCTTCCAAATGTGGA-3′. Guide RNAs and ssDNAs were injected into fertilized embryos (F0 mutant animals or founders). F0 embryos were transferred into pseudopregnant mice. Founders were bred with C57BL/6N mice to generate F1 mice.

### Genotyping

Ear biopsies were collected and incubated overnight at 55°C while shaking at 800 rpm in a proteinase K-lysis buffer (200 mM NaCl, 100 mM Tris pH 8.5, 5 mM EDTA, 0.2% SDS). PCRs were performed using supernatant of the lysis preparation as DNA template (20–100 ng), 1X Taq PCR buffer, 2 mM MgCl_2_, 400 µM dNTPs, 1.25 U Taq-polymerase (Thermofisher Scientific) and 0.5 µM of primers. A 499 bp fragment of *Piezo2* locus was amplified using the forward 5′-GAAAGAGCTACTTTGAAAGGAGTATGTGC-3′ and reverse 5′-CCTGTCAGAAGAGAAATGGTTGCC-3′ primers. Inserted point mutations generated new restrictions sites that allow the identification of wild-type, heterozygous and homozygous animals from the knock-in mice. PCR products were incubated overnight with BspI and MseI restriction endonucleases (NEB Inc.) for *Piezo2^R2756H^* and *Piezo2^R2756K^* mouse lines, respectively. Amplified and digested DNA fragments were observed by gel electrophoresis.

### RNAscope

Lumbar DRGs were collected from adult animals and incubated for 40 min in Zamboni's fixative media (2% paraformaldehyde + picric acid), washed with PBS and incubated in 30% sucrose (in PBS) overnight at 4°C. DRGs were embedded in OCT Tissue Tek (Sakura). Ten-micrometre thick cryosections were stored at −80°C until used for experiments. *In situ* hybridization was carried out according to the manufacturer's instructions (RNAscope^TM^ Multiplex Fluorescent V2 assay, ADC, Kit No. 323110, *Piezo2* probe No. 4001191). LSM700 Carl Zeiss and CSU-WI Olympus spinning disk confocal microscopes were used to acquired images at 20× and numerical apertures 0.5 and 0.8, respectively. Fluorescence intensity was analysed using Fiji.^22^

### Behavioural testing

All details of the behavioural test used can be found in the Supplementary material.

### Statistical analysis

All data analyses were performed using GraphPad Prism and all data sets were tested for normality. Parametric data sets were compared using a two-tailed, Student's *t*-test. Non-parametric data sets were compared using a Mann–Whitney test. To compare more than two groups, one- or two-way ANOVA analyses were used. Categorical data were compared using χ^2^ tests.

## Results

### Mutations related to human diseases in PIEZO2 are gain-of-function

**Figure 2:**
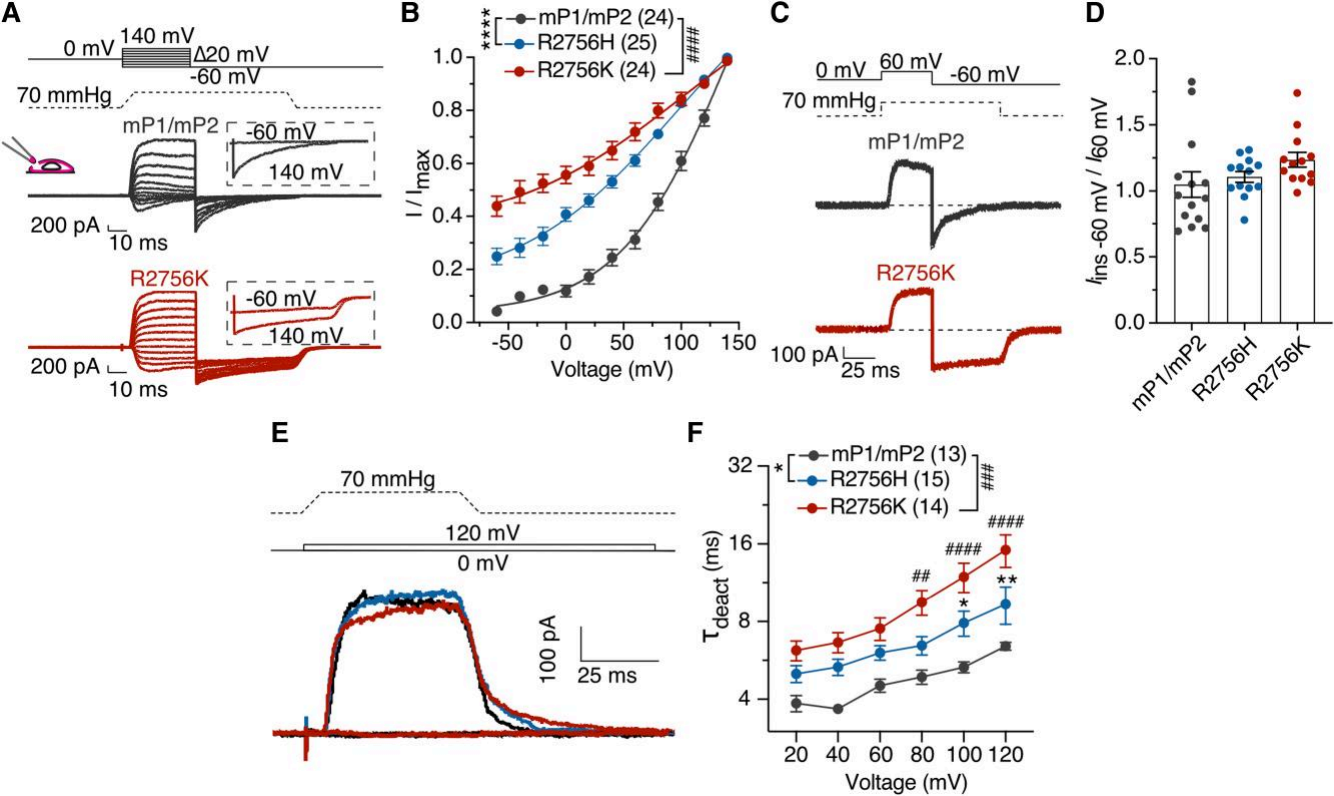
**Voltage-block is released in Piezo2 variants.** (**A**) Representative traces of the tail current protocol performed in N2a*^Piezo1−/−^* cells expressing the chimeric channels. *Inset*: Tail currents evoked after pre-stimuli of −60 and 140 mV. (**B**) The apparent open probability increased in the mutants at physiological pulses. Tail currents were normalized to their maximum (two-way ANOVA, Sídák test; ****P < 0.0001, ^####^*P* < 0.0001). (**C**) Example traces of the rectification index (*I*_ins__−60_ _mV_/*I*_60_ _mV_) protocol. (**D**) Rectification index is similar in the mutants. (**E**) Representative traces from the deactivation protocol in the chimeric channel variants. (**F**) Mutants displayed slower deactivation kinetics at depolarizing pulses. An exponential fit was calculated to measure the deactivation time (τ_deact_; two-way ANOVA, Sídák test; *P < 0.05, **P = 0.007, ^##^*P* = 0.002, ^###^*P* = 0.0002, ^####^*P* < 0.0001). Data are presented as mean ± standard error of the mean.

We first asked whether the conserved R2756 residue also controls the voltage sensitivity of mPIEZO2 channels. We thus generated *mPiezo2* channels with single missense mutations (R2756H, R2756C and R2756K), known to be associated with human developmental diseases.^15,16^ We first quantitatively assessed mechanosensitivity using substrate deflection of N2a*^Piezo1−/−^* cells expressing wild-type or mutant *Piezo2* channels^12,17,18,23^ (Fig. 1A and Supplementary Fig. 1). We measured three types of mechanically-gated currents in cells expressing *Piezo2* channels: rapidly adapting (RA), intermediate adapting (IA) and slowly adapting currents (SA) (Fig. 1B and Supplementary Fig. 1D). Cells expressing the R2756H, R2756C and R2756K mutations exhibited significantly fewer RA and increased proportions of IA and SA currents compared to wild-type (Fig. 1B and Supplementary Fig. 1D). The deflection-current relationship revealed that R2756K mutant channels are more sensitive to pili deflection compared to wild-type or R2756H/R2756C mutant channels (Fig. 1C and Supplementary Fig. 1E). Consistently the mean deflection threshold for R2756K was almost 5-fold lower than that of wild-type or R2756H/R2756C mutant channels (Fig. 1D and Supplementary Fig. 1F). We also noted subtle, but significant changes in the kinetics of mechanosensitive currents generated by *Piezo2* mutant channels (e.g. small increase in latency for activation) (Supplementary Tables 1 and 2). We next measured the effects of these mutations on the stretch and voltage sensitivity of PIEZO2 channels. Mutations were introduced into the stretch-sensitive chimeric channel mP1/mP2^12^ and currents were measured from excised outside-out patches. The R2756K and R2756H mutant channels displayed significantly enhanced stretch sensitivity compared to wild-type chimeric channels, but the R2756C substitution did not alter stretch sensitivity (Fig. 1E and Supplementary Fig. 2A and B). Additionally, the R2756K and R2756H chimeric variants showed significantly slower inactivation kinetics to membrane stretch compared to wild-type channels (Supplementary Fig. 2D). Indentation-induced currents were also examined in N2a*^Piezo1−/−^* cells expressing wild-type or mutant *Piezo2* channels and consistent with the results from pili experiments, R2756K and R2756H mutant channels displayed RA-currents with lower thresholds for activation compared to wild-type (Fig. 1F and G). We also found that indentation-induced RA-currents showed slower inactivation kinetics compared to wild-type which reached statistical significance for the R2756H mutation (Supplementary Fig. 2F and G), similar to results from another *Piezo2* mutation associated with distal arthrogryposis.^24^ We next used a tail current protocol to measure channel availability in outside out patches subjected to rapid pressure pulses^12^ (Fig. 2A). Between 25% and 45% of the maximum tail current could be measured from the R2756H and R2756K chimeric variants at −60 mV compared to less than 5% in wild-type (Fig. 2A and B and Supplementary Fig. 3). Thus, both R2756H and R2756K mutations substantially relieve the voltage-block of these chimeric channels at physiological membrane potentials. The effect on the tail current was not accompanied by any change in the rectification index (Fig. 2C and D). PIEZO2 channels inactivate very rapidly at negative potentials making it challenging to study deactivation kinetics. We thus measured the effects of pressure removal at a series of positive voltages on current deactivation and found that R2756H and R2756K chimeric variants showed significantly slower deactivation compared to the wild-type chimera (Fig. 2E and F). A considerable delay in channel closing was also observed during the transition from the inactivated to deactivated state after pressure removal (Supplementary Fig. 3B and D). In conclusion, slower inactivation and deactivation, increased mechanosensitivity and an almost complete removal of voltage-block were the main effects of the R2756H and R2756K *mPiezo2* missense mutations, with the R2756K mutation clearly displaying the strongest effects on mechanosensitivity.

### Subpopulations of mechanoreceptors are mildly sensitized in *Piezo2* knock-in mice

**Figure 3:**
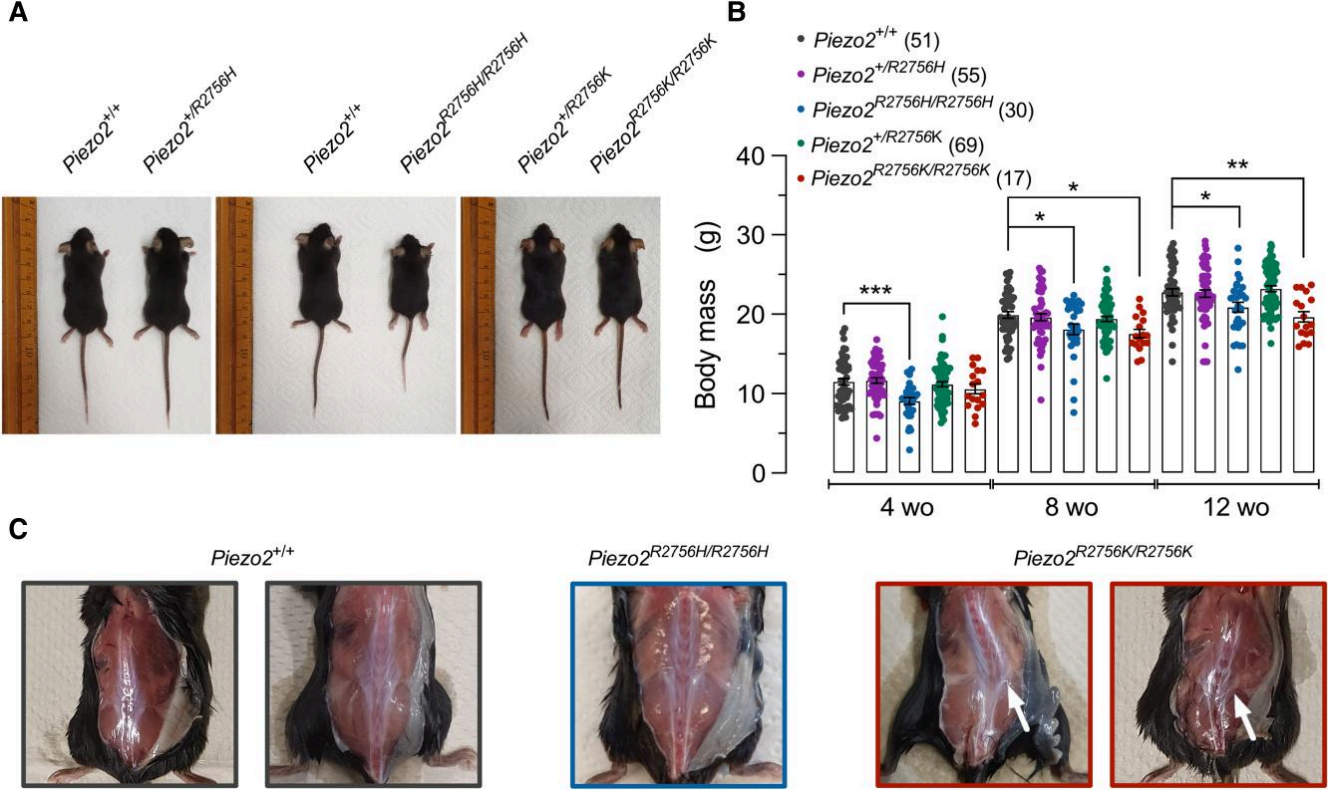
**Pathogenic mutation in Piezo2 showed reduced weight and scoliosis in knock-in mice.** (**A**) Photos of knock-in mice at 5 weeks of age. Note that *Piezo2^R2756H/R2756H^* mice are smaller than controls. (**B**) Bar plot showing that *Piezo2^R2756H/R2756H^* mice are smaller at Week 4 after birth and that both *Piezo2^R2756H/R2756H^* and *Piezo2^R2756K/R2756K^* showed reduced weight at Weeks 8 and 12. Each dot represents an animal (mean ± standard error of the mean; one-way ANOVA; *P < 0.05, **P < 0.01, ***P = 0.0003). (**E**) Pictures of *Piezo2^+/+^*, *Piezo2^R2756H/R2756H^* and *Piezo2^R2756K/R2756K^* mice. Of 24 *Piezo2^R2756K/R2756K^* animals examined, 12 showed scoliosis.

Our biophysical measurements led us to predict that introduction of R2756H and R2756K into the mouse genome should radically alter the mechanosensitivity of endogenous PIEZO2-dependent currents. We generated two knock-in mouse lines that globally express the R2756H and R2756K variants (*Piezo2^R2756H^* and *Piezo2^R2756K^* mice) (Supplementary Fig. 4A and B). The orthologous human mutation of *Piezo2^R2756H^* has been associated with short stature and scoliosis.^15,16,25^ Interestingly, we found that homozygous *Piezo2^R2756H/R2756H^* animals weighed on average ∼20% less than wild-type controls at 4 weeks of age. At 8 and 12 weeks of age, both *Piezo2^R2756H/R2756H^* and *Piezo2^R2756K/R2756K^* mice weighed significantly less on average than wild-types (∼9% less), however, this effect was only partially penetrant as many of the mutant mice had body weights in the same range as controls. No effect of the mutations on body weight was observed in heterozygous animals (Fig. 3A and B). In ∼50% of the *Piezo2^R2756K/R2756K^* mice (12/24) we observed abnormal spine curvature (scoliosis), but this phenotype was not observed in heterozygotes or in *Piezo2^R2756H/R2756H^* mutant mice (Fig. 3C). Unlike mice with a constitutive gain-of-function mutation (E2727del) that also slows Piezo2 inactivation,^24^ we never observed joint abnormalities reminiscent of distal arthrogryposis in our mice.

**Figure 4:**
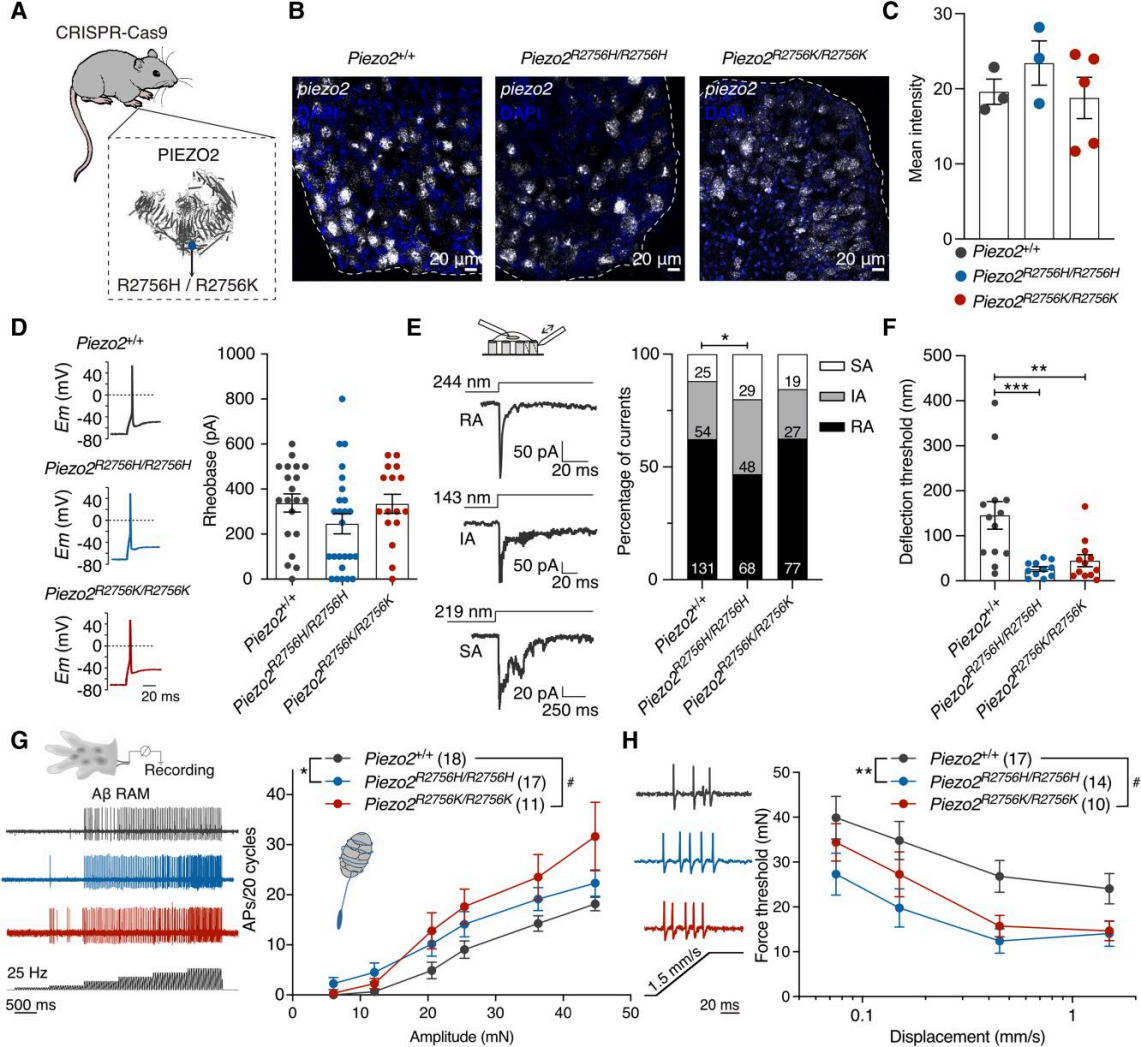
**Mechanoreceptors from knock-in mice are more sensitive to deflection stimuli.** (**A**) Cartoon representing the global insertion of the mutations in *Piezo2* knock-in mice. (**B**) Representative images of *Piezo2 in situ* hybridization (RNAscope) in lumbar dorsal root ganglia (DRG). White, *Piezo2* mRNA; blue, 4′,6-diamidino-2-phenylidole (DAPI). Dashed lines show the limits between the DRG section and background. (**C**) Quantification of area of transcript fluorescence from all sections. Each dot represents the mean value from each mouse (Kruskal–Wallis test; *P* > 0.05). (**D**) *Left*: Representative action potentials (APs) in mechanoreceptors from *Piezo2*^+/+^, *Piezo2^R2756H/R2756H^* and *Piezo2^R2756K/R2756K^* animals. *Right*: Distribution of AP thresholds (rheobase) showing no differences between mutants and wild-type. (**E**) *Left*: Representative traces of the three types of deflection-gated currents [rapidly adapting (RA), intermediate adapting (IA) and slowly adapting (SA)] from *Piezo2*^+/+^ mechanoreceptors. *Right*: Histograms showing that *Piezo2^R2756H/R2756H^* neurons displayed less RA currents compared to wild-type cells (χ^2^ test; *P = 0.01). Numbers indicate the total of currents recorded. Note scale differences in the representative traces. (**F**) Deflection thresholds were lower in *Piezo2^R2756H/R2756H^* and *Piezo2^R2756K/R2756K^* cells compared to wild-type (Kruskal–Wallis test; **P = 0.008, ***P = 0.0008). (**G**) *Left*: Example traces of single rapidly-adapting mechanoreceptor (RAM) amyloid-β (Aβ) fibres form wild-type and *Piezo2* knock-in mice in response to a 25 Hz vibration stimulus. *Right*: Increased AP firing was observed in RAM Aβ fibres from the mutants compared to wild-type (two-way ANOVA with Sídák *post hoc* analysis; *P = 0.04, ^#^*P* = 0.01). (**H**) *Left*: Representative ramp responses of individual RAMs from *Piezo2* knock-in and wild-type mice at 1.5 mm/s. *Right*: Displacement-thresholds relationships showing that RAM Aβ fibres from Piezo2 mutants are more sensitive to mechanical stimuli (two-way ANOVA with Sídák *post hoc* analysis; **P = 0.007, ^#^*P* = 0.03). Data are presented as mean ± standard error of the mean.

The introduction of missense mutations could alter gene expression, we thus examined *Piezo2* expression in sensory neurons within the DRG using RNAscope. We found that in the DRG *Piezo2^+/+^*, *Piezo2^R2756H/R2756H^* and *Piezo2^R2756K/R2756K^* mice showed similar *Piezo2* mRNA levels (Fig. 4B and C).

**Figure 5:**
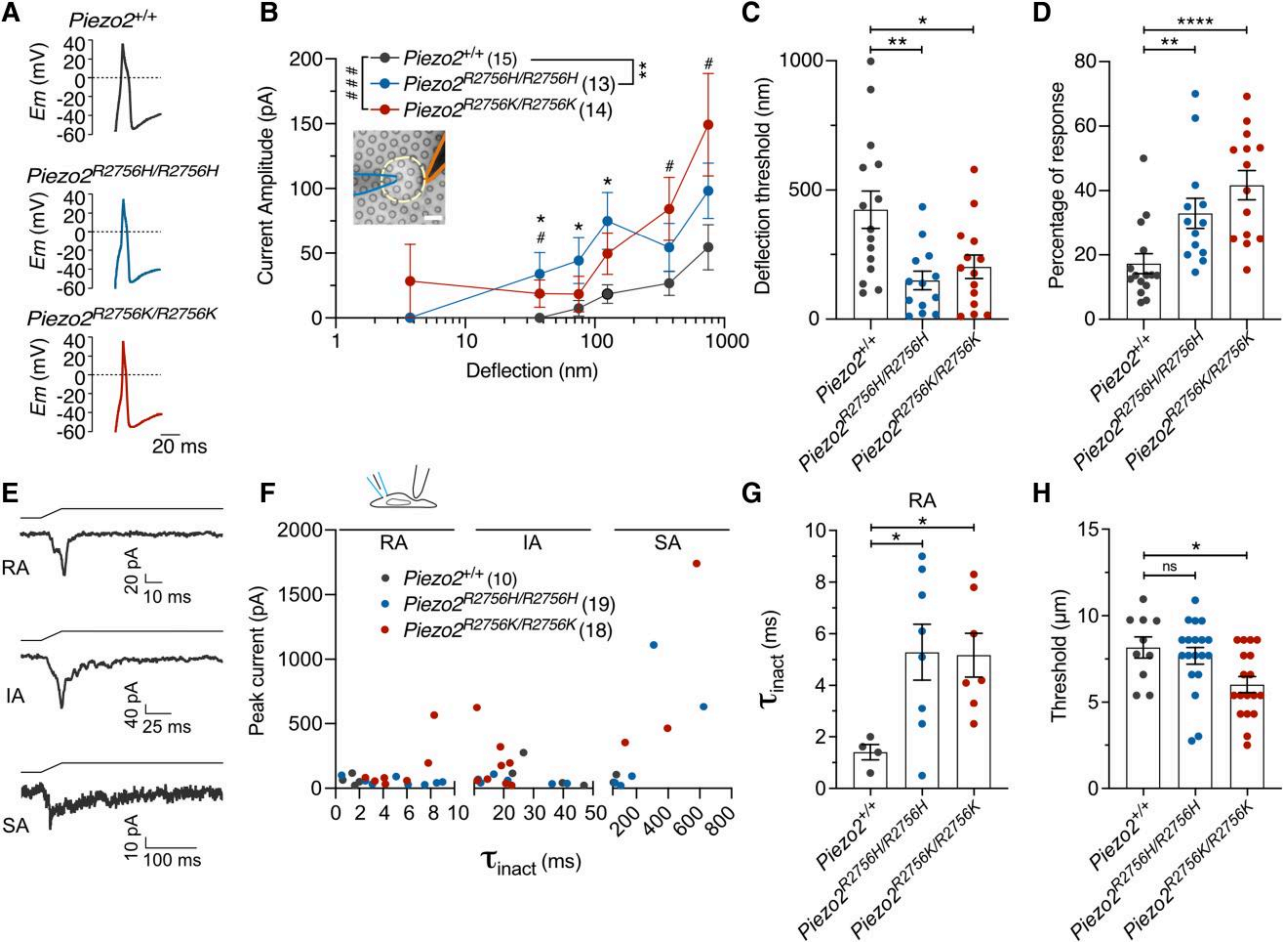
**Nociceptors from *Piezo2* knock-in mice showed mechanical hypersensitivity.** (**A**) Representative action potentials (APs) in nociceptors from *Piezo2*^+/+^, *Piezo2^R2756H/R2756H^* and *Piezo2^R2756K/R2756K^* animals. (**B**) Deflection-current amplitude relationship of nociceptors showing that neurons from knock-in mice displayed hypersensitive deflection-gated currents. (Mann–Whitney test; *P < 0.05, ^#^*P* < 0.05; additionally, an ordinary two-way ANOVA indicated differences between wild-type and the mutants; **P = 0.005; ^###^*P* = 0.0008). (**C**) Deflection thresholds were lower in nociceptors from knock-in mice compared to wild-type (one-way ANOVA; *P = 0.01, **P = 0.002). (**D**) Histogram showing that nociceptors from *Piezo2^R2756H/R2756H^* and *Piezo2^R2756K/R2756K^* are more responsive to deflection stimuli compared to controls (Kruskal–Wallis test; **P = 0.005, ****P < 0.0001). (**E**) Representative recordings of the three types of mechanosensitive currents from isolated sensory neurons using the indentation method [rapidly adapting (RA), intermediate adapting (IA) and slowly adapting (SA) currents]. (**F**) τ_inact_—peak current relationship from mechanosensitive currents on isolated nociceptors using the indentation method. (**G**) Histogram showing that isolated nociceptors from *Piezo2* knock-in mice displayed RA currents with longer inactivation kinetics compared to wild-type (Kruskal–Wallis test; *P < 0.05) Each dot represents the inactivation kinetics value from the last stimulus applied in each cell. (**H**) Nociceptors from *Piezo2^R2756K/^*^R2756K^ mice showed lower apparent mechanical threshold compared to control neurons using the indentation method (Kruskal–Wallis test; *P = 0.02).

In the complete absence of *Piezo2*, around half of mechanoreceptors are completely insensitive to mechanical stimuli.^2,5^ We next recorded mechanosensitive currents in wild-type and mutant sensory neurons in culture which had been classified as mechanoreceptors or nociceptors according to their size and action potential (AP) shape as previously reported^17,26-29^ (Figs 4D and 5A). When performing current clamp recordings from mechanoreceptors we found their excitability to be unaffected by the *Piezo2* point mutations as reflected by unaltered resting membrane potentials, rheobase or input resistance (Fig. 4D, Supplementary Fig. 5A and Supplementary Table 3). We next recorded deflection gated currents from mechanoreceptors and again identified mechanically activated currents with RA, IA and SA kinetics, with RA-currents predominating.^17,29^ Mechanoreceptors from *Piezo2^R2756H/R2756H^* and *Piezo2^+/R2756K^* showed a small but significant decrease in the proportion of RA-currents compared to wild-type cells, but no significant differences were observed in mechanoreceptors from *Piezo2^R2756K/R2756K^* and *Piezo2^+/R2756H^* mice (Fig. 4E and Supplementary Fig. 6B). Deflection-current amplitude relationships were similar between genotypes with a trend for mechanoreceptors from *Piezo2^R2756K/R2756K^* mice to show higher sensitivity (Supplementary Fig. 6B and D). However, we observed robust and statistically significant reductions in the mean minimum deflection amplitudes capable of evoking mechanosensitive currents in all homozygous and heterozygous variant genotypes compared to wild-type (Fig. 4F and Supplementary Fig. 6E). This change in threshold was accompanied by small changes in the kinetic parameters of mechanosensitive currents, for example in the inactivation kinetics of RA-currents in *Piezo2^R2756H/R2756H^* mutants (Supplementary Table 3). We also examined native indentation-induced currents in isolated mechanoreceptors from wild-type and *Piezo2^R2756H/R2756H^* or *Piezo2^R2756K/R2756K^* mice. But, in contrast to wild-type mechanoreceptors, many indentation currents (∼30%–50%) measured in *Piezo2* mutants showed slowed inactivation so that they were classified as IA currents (Supplementary Fig. 7A–C and Supplementary Table 4).

We next asked if the threshold for gating mechanosensitive currents in isolated sensory neurons was accompanied by changes in the properties of intact mechanoreceptors. Using an *ex vivo* preparation we recorded from single mechanoreceptors innervating the hind paw glabrous skin.^20,21^ We found that rapidly-adapting mechanoreceptors (RAMs) from *Piezo2^R2756H/R2756H^* and *Piezo2^R2756K/R2756K^* mutants that innervate Meissner's corpuscles displayed mildly enhanced firing to small 25 Hz sinusoidal stimuli compared to wild-type (Fig. 4G). During the ramp phase of the mechanical stimulus RAMs recorded from *Piezo2^R2756H/R2756H^* and *Piezo2^R2756K/R2756K^* fired with shorter latencies reflecting lower force thresholds that were up to 10 mN smaller than wild-type mice (∼50% reduction) (Fig. 4H and Supplementary Fig. 8A and B). In contrast, slowly-adapting mechanoreceptors (SAMs) associated with Merkel cells^30,31^ were barely affected by either missense mutation (Supplementary Fig. 8C–F). Thus, a subpopulation of mechanoreceptors had significantly altered receptor properties when the biophysical properties of PIEZO2 are altered. This data is consistent with the idea that other mechanosensitive channels may underlie mechanoreceptor function.^8^

### Nociceptors from *Piezo2^R2756H^* and *Piezo2^R2756K^* mice showed mechanical hypersensitivity

We were surprised by the fact that large changes in the biophysical properties of endogenous PIEZO2 channels only had mild effects on touch receptors. However, there is increasing evidence that PIEZO2 may also play a role in pain sensitivity.^5,7,32^ Cutaneous nociceptors that detect intense mechanical stimuli do not lose mechanosensitivity in the absence of PIEZO2, but show reduced initial firing to step mechanical stimuli.^5^*Piezo2* is expressed by most nociceptors and so we next examined the effects of *Piezo2* missense mutations on nociceptor physiology. We measured the mechanosensitivity of nociceptive sensory neurons in culture with broad humped action potentials (Fig. 5A). We found that the deflection evoked currents were often three times larger at all deflection amplitudes in neurons from *Piezo2^R2756H/R2756H^* and *Piezo2^R2756K/R2756K^* mice compared to wild-type cells (Fig. 5B). In addition, the threshold for current activation was substantially lowered to values typical of mechanoreceptors in both types of mutant neurons (Figs 4F and 5C). The frequency with which a mechanical stimulus evoked currents was also substantially increased in mutant neurons compared to wild-type (Fig. 5D). We also noted significant, but milder, increases in the sensitivity of deflection evoked currents in neurons from animals in which either mutation was present on only one allele (Supplementary Fig. 6A–C). Again, we repeated this analysis using indentation stimuli on isolated nociceptors from wild-type and both *Piezo2^R2756H/R2756H^* and *Piezo2^R2756K/R2756K^* mice and observed RA currents with significantly increased inactivation kinetics compared to wild-type (Fig. 5E–G and Supplementary Table 6). The mean threshold for indentation-induced currents was also lower in nociceptors *Piezo2^R2756K/R2756K^* mice compared to wild-type (Fig. 5H).

Normally, acutely cultured sensory neurons exhibit little or no ongoing action potential firing.^33-35^ Interestingly, we found that nociceptors from *Piezo2^R2756H/R2756H^* and *Piezo2^R2756K/R2756K^* often exhibited ongoing firing in the absence of current injection compared to wild-type neurons (Supplementary Fig. 10D and E). Using indentation stimuli in current clamp mode we noted that the mechanical thresholds for action potential initiation were lower in nociceptors from Piezo2 point mutant mice and this was statistically different for *Piezo2^R2756H/R2756H^* mice compared to wild-type (Supplementary Fig. 10F). Moreover, we measured the rheobase of nociceptor neurons from *Piezo2^R2756H/R2756H^* and *Piezo2^R2756K/R2756K^* animals and found this to be decreased by 30% and 55%, respectively, compared to wild-type (Supplementary Fig. 11A and B). Such changes in electrical excitability could be due to alterations in voltage-gated conductances; however, direct measurements of macroscopic voltage-gated inward and outward currents revealed no significant differences between wild-type and mutant neurons (Supplementary Figs 5B–D and 11C and D). The resting membrane potential of mutant neurons was also not altered compared to wild-type (Supplementary Tables 5 and 6). Sensory neurons in culture display spontaneous membrane potential fluctuations the amplitudes of which are partially dependent on voltage gated sodium channels.^34^ We also observed membrane potential fluctuations in sensory neurons from wild-type and *Piezo2^R2756H/R2756H^* and *Piezo2^R2756K/R2756K^* mice, but they did not differ between genotypes (Supplementary Fig. 11E and F). Thus, nociceptors from *Piezo2^R2756H/R2756H^* and *Piezo2^R2756K/R2756K^* mice exhibit substantial mechanical hyperexcitability.

### C-fibres in *Piezo2* knock-in mice displayed spontaneous firing

**Figure 6:**
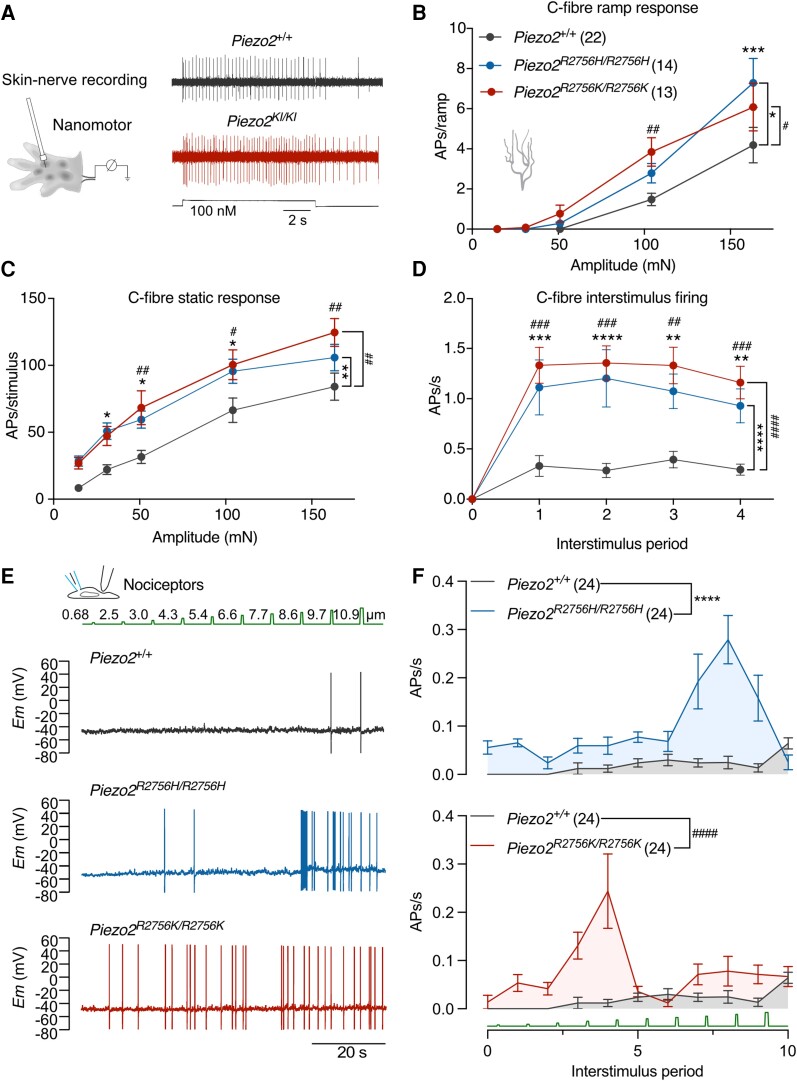
**C-fibres from knock-in mice showed spontaneous action potential firing after removal of mechanical stimuli.** (**A**) *Left*: Cartoon representing *ex vivo* glabrous skin-nerve preparation. *Right*: Representative traces of C-fibre activity during a 100 mN stimulus from control (*Piezo2*^+/+^) and *Piezo2* knock-in (*Piezo2^KI/KI^*) animals. (**B**–**D**) Spike activity during ramp phase (**B**, two-way ANOVA with Sídák *post hoc* analysis; *P = 0.02, ^#^*P* = 0.02, ^##^*P* = 0.007, ***P < 0.001), static phase (**C**, two-way ANOVA with Sídák *post hoc* analysis; *P < 0.05, ^#^*P* < 0.05, **P = 0.003, ^##^*P* < 0.01) and interstimulus firing (**D**, two-way ANOVA with Sídák *post hoc* analysis; **P < 0.01, ^##^*P* < 0.01, ***P < 0.001, ^###^*P* < 0.001, ****P < 0.0001, ^####^*P* < 0.0001). Data are presented as mean ± standard error of the mean. (**E**) Representative current-clamp recordings of action potential (AP) firing elicited by mechanical stimulation using the indentation method on isolated nociceptors from *Piezo2*^+/+^, *Piezo2^R2756H/R2756H^* and *Piezo2^R2756K/R2756K^* animals. (**F**) Interstimulus period-APs/s relationship showing an enhanced AP firing on nociceptors from *Piezo2* knock-in mice after removal of mechanical stimuli compared to controls (two-way ANOVA; ****P < 0.0001, ^####^*P* < 0.0001).

**Figure 7:**
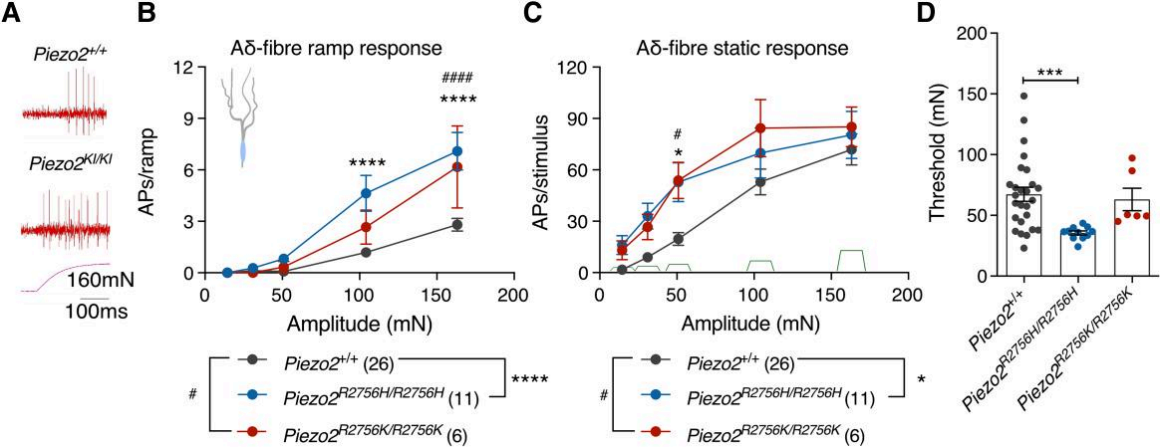
**Aδ-nociceptor firing is enhanced in *Piezo2* knock-in mice.** (**A**) Example traces of responses to ramp phases in Aδ-nociceptor from *Piezo2^+/+^* and *Piezo2* knock-in animals during a 160 mN stimulus. (**B**) Aδ-fibre firing activity during ramp phase (two-way ANOVA with Sídák *post hoc* analysis; *P < 0.05, ****P < 0.0001, ^####^*P* < 0.0001). Note that both mutants showed enhanced firing during the dynamic phase compared to controls. (**C**) Static phase responses from Aδ-fibres in *Piezo2^+/+^* and mutants (two-way ANOVA with Sídák *post hoc* analysis; *P < 0.05, ^#^*P* < 0.05). Knock-in mice displayed higher firing activity at the 31 mN force compared to controls. (**D**) Dot plot showing that Aδ-nociceptors from *Piezo2^R2756H/R2756H^* are more sensitive to mechanical stimuli compared to controls (Kruskal–Wallis; ***P < 0.001). Data are presented as mean ± standard error of the mean.

We next examined intact nociceptors innervating the skin of which there are two main classes, thinly myelinated Aδ-fibre or unmyelinated C-fibre nociceptors.^36^ In the glabrous skin we observed using quantitative force stimuli that C-fibre nociceptors from *Piezo2^R2756H/R2756H^* and *Piezo2^R2756K/R2756K^* mice exhibited substantially increased firing rates and much lower mechanical thresholds for activation compared to wild-type (Fig. 6A–D) as did Aδ-fibre mechanonociceptors (Fig. 7 and Supplementary Fig. 1^2^). We analysed firing during stimulus onset (ramp phase) separately from the static phase and found that there was a substantial sensitization to both phases in C-fibre and Aδ-fibre mechanonociceptors in both mutant mice (Figs 6B, C and 7 and Supplementary Figs 12 and 13). A hallmark feature of C-fibre nociceptors, first described by Perl in the 1960s, is that they often continue to fire after the noxious mechanical stimulus is removed.^37^ Strikingly, C-fibres recorded from the mutants showed substantially increased ongoing firing after removal of mechanical stimuli compared to wild-type C-fibres (Fig. 6D). We quantified this change in C-fibres from *Piezo2^R2756H/R2756H^, Piezo2^+/R2756K^, and Piezo2^R2756K/R2756K^* mice and found that in these genotypes C-fibres exhibited up to three-fold increased interstimulus firing activity compared to wild-type (Fig. 6D and Supplementary Fig. 13C). Only in heterozygous *Piezo2^+/R2756H^* mice, was the interstimulus firing equivalent to that seen in wild-type controls.

Our finding that mechanical stimuli in the intact skin induced ongoing activity led us to hypothesize that mechanical stimulation of isolated nociceptors in culture may have initiated the spontaneous activity (Supplementary Fig. 10D and E). We designed the following experiment to test this idea, isolated nociceptors were recorded in current clamp mode and confronted with series of poking stimuli of increasing size over a period of 90 s (Fig. 6E). We monitored spiking activity and noted that even in wild-type cells a very low level of spontaneous activity was initiated following the mechanical stimuli (Fig. 6E and F). In contrast, low levels of spontaneous activity were observed in nociceptors from *Piezo2^R2756H/R2756H^ and Piezo2^R2756K/R2756K^* mice before the mechanical stimuli were applied (Fig. 6F). We quantified spike rates over the entire population, excluding spikes initiated directly by the mechanical stimulus (Fig. 6F). Interestingly, there was a clear trend for mechanical stimuli to increase spontaneous rates of firing, especially in cells from *Piezo2^R2756K/R2756K^* mice (Fig. 6F), a finding consistent with the idea that once opened mutant channels may remain open and heighten membrane excitability. These *in vitro* and *ex vivo* data show that voltage control of PIEZO2 channels in nociceptors is crucial for conferring high mechanical thresholds to mammalian nociceptors. Furthermore, the impairment in the ability of mutant channels to deactivate after opening was correlated with a large increase in ongoing activity of nociceptors in the absence of a mechanical stimulus.

### *Piezo2* knock-in mice showed enhanced mechanical pain *in vivo*

**Figure 8:**
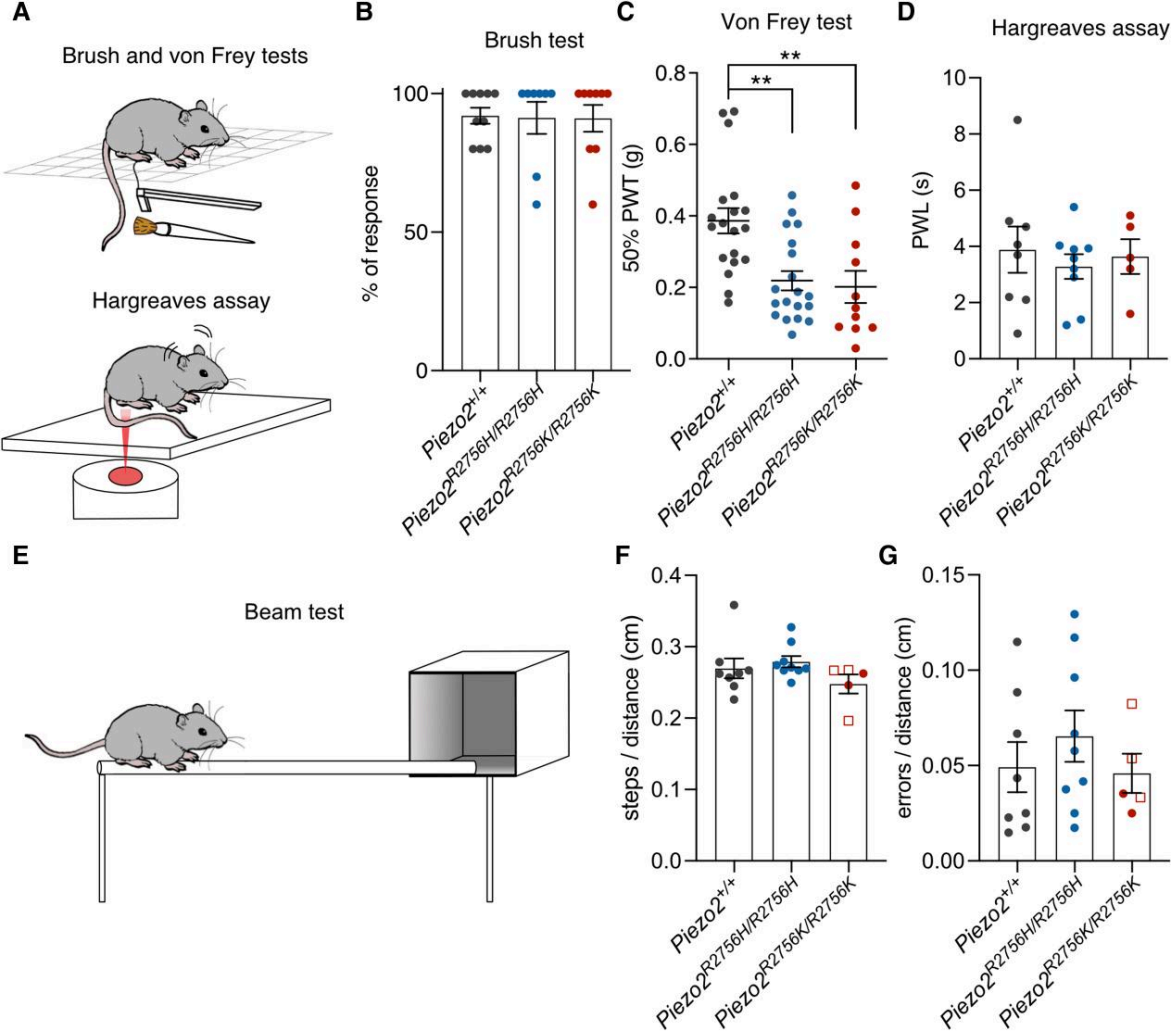
**Mechanical pain hypersensitivity in *Piezo2* knock-in mice.** (**A**) Cartoon representing the brush, von Frey and Hargreaves behaviour assays. (**B**) Histogram showing the percentage of response to brush stimulation in mutants (*Piezo2^R2756H/R2756H^*, *n* = 8; *Piezo2^R2756K/R2756K^*, *n* = 9) and wild-type (*n* = 10). (**C**) *Piezo2^R2756H/R2756H^* (*n* = 19) and *Piezo2^R2756K/R2756K^* (*n* = 11) animals showed a reduced (50%) paw withdrawal threshold (PWT) compared to controls (*n* = 19) (one-way ANOVA; **P = 0.001). Each dot represents average values from different measures taken on different days in each animal. (**D**) *Piezo2* knock-in mice exhibited similar responses to thermal pain sensation (*Piezo2^+/+^ n* = 8*; Piezo2^R2756H/R2756H^*, *n* = 9; *Piezo2^R2756K/R2756K^*, *n* = 5). PWL = paw withdrawal latency. (**E**) Scheme representing the beam test. (**F** and **G**) *Piezo2* knock-in mice did not show proprioceptive deficits (steps/distance and errors/distance relationships) when tested under the beam assay. Empty squares indicate post-mortem examined *Piezo2^R2756K/R2756K^* mice that developed scoliosis. Data are presented as mean ± standard error of the mean.

Apart from a non-penetrant scoliosis or occasional growth retardation, especially in *Piezo2^R2756K/R2756K^* mice (Fig. 3), the *Piezo2* knock-in mice appeared largely healthy, with no obvious motor deficits (Fig. 8E–G). We tested behavioural responses to innocuous brushing of the hind paw and found no obvious hypersensitivity in *Piezo2^R2756H/R2756H^* and *Piezo2^R2756K/R2756K^* mice compared to wild-type controls (Fig. 8A and B). However, paw withdrawal to punctate stimulation was clearly sensitized with paw withdrawal thresholds (PWT)^38-40^ on average half those of controls in both *Piezo2^R2756H/R2756H^* and *Piezo2^R2756K/R2756K^* mutant genotypes (Fig. 8C). The nociceptor hyperexcitability to mechanical stimuli likely underlies behavioural hypersensitivity to punctate stimuli, but some of the same nociceptive neurons can also signal noxious heat.^41^ Thus we also measured behavioural withdrawal latencies to noxious heat using the Hargreaves test,^42^ but interestingly found no differences between wild-type and both *Piezo2* point mutants (Fig. 8D). We assessed motor coordination using elevated beam and ladder tests as well as a gait analysis using the mouse walker system (Fig. 8E–G, Supplementary Fig. 14A–C and Supplementary Table 7). Both *Piezo2^R2756H/R2756H^* and *Piezo2^R2756K/R2756K^* mice performed no different from wild-type mice in beam and ladder walking tasks (Fig. 8E–G and Supplementary Fig. 14A–C). Interestingly, it was also clear that even *Piezo2^R2756K/R2756K^* animals found to have scoliosis at sacrifice did not perform any worse than unaffected littermates in beam and ladder walking (Fig. 8E–G and Supplementary Fig. 14A–C). Gait analysis revealed largely normal locomotion in both mutants with the exception that *Piezo2^R2756H/R2756H^* animals which displayed a slight deficit in their ability to walk in a straight line (Supplementary Table 7). Finally, we found that our *Piezo2* point mutation mice showed no signs of enhanced spontaneous pain as assessed using a naturalistic digging assay (Supplementary Fig. 14D).^43^ In summary, the loss of voltage-block of Piezo2 is associated with a specific enhancement of mechanical pain sensitivity *in vivo*, without accompanying effects on motor coordination or touch.

## Discussion

Here we have shown that the biophysical properties of PIEZO2 channels sets the sensitivity and mechanical thresholds of nociceptors required to detect painful mechanical stimuli. Changing PIEZO2 residue R2756 to histidine or lysine made nociceptors approximately 3-fold more sensitive to mechanical stimuli with mechanical thresholds similar to low threshold mechanoreceptors. In contrast to nociceptors, changing the biophysical properties of PIEZO2 was associated with only minor changes in the threshold and suprathreshold sensitivity of some, but not all touch receptors. Remarkably, single missense mutations in *Piezo2* were sufficient to induce ongoing activity and sensitization of nociceptors *in vivo*. Indeed, direct microneurographic recordings from human C-fibres have shown that patients with a variety of painful conditions including painful small fibre neuropathy, painful diabetic neuropathy, and fibromyalgia display marked ongoing activity in C-fibres as well as sensitization.^44-50^ The fact that the mutations here relieve voltage block of the PIEZO2 channels strongly suggests that physiological or pathological sensitization of nociceptors partially requires PIEZO2 channels. Our findings provide a clear mechanistic explanation for the deficits in pain hypersensitivity seen in mice lacking PIEZO2 channels in sensory neurons.^5,32^ Furthermore, our data illustrate how hyperexcitability of a mechanically-gated channel can in principle be sufficient to support ongoing activity in nociceptors that is associated with chronic pain (Supplementary Fig. 1^5^).

*Piezo2* is required for normal proprioception in mice and humans.^1,51,52^ It has also been suggested that loss of PIEZO2 function in proprioceptors may alone be sufficient to cause skeletal abnormalities in mice.^53^ Indeed, human skeletal abnormalities are symptomatic of both gain- and loss-of-function mutations in human *PIEZO2*. One previous study examined mice with another gain-of-function *Piezo2* mutation (mouse E2799del) which similarly to the mutations examined in this study, slowed the inactivation properties of PIEZO2 channels.^24^ Homozygous *Piezo2^E2799del^* mice apparently developed hindlimb contractures, a phenotype that we did not observe in *Piezo2^R2756K/R2756K^*or *Piezo2^R2756H/R2756H^* mice. We did, however, observe a partially penetrant spinal scoliosis phenotype, but only in mice carrying the R2756K mutation (Fig. 3), however, neither of our knockin mouse mutants showed significant proprioceptive deficits as indicated by behavioural assessments of motor coordination (Fig. 8 and Supplementary Fig. 14). It thus remains unclear why some *Piezo2* gain-of-function mutations in mice are associated with skeletal abnormalities and others not. The discrepancy may be due to differences in the way different mutations affect the physiology of proprioceptors. However, Ma and colleagues^24^ recorded mechanically gated currents in unidentified cultured sensory neurons and did not record from identified mechanoreceptors or proprioceptors in their *Piezo2* gain-of-function mice, making definitive conclusions difficult. Skeletal abnormalities are nevertheless seen in patients carrying the same orthologous R2756H mutation that we studied here in mice,^15,16^ thus there may be species specific factors affecting the penetrance of skeletal phenotypes associated with PIEZO2.

We were surprised to find that both mechanically-gated currents in touch receptors and mechanoreceptor function were only partially affected by Piezo2 gain-of-function mutations. However, this finding is consistent with recent findings that another mechanically-gated ion channel ELKIN1 is required for touch receptor function in addition to PIEZO2.^8,19^ It remains puzzling, however, why rare patients with complete *PIEZO2* loss-of-function alleles appear to be completely touch insensitive.^1^ However, *PIEZO2* mutations are associated with a variety of human developmental disorders, including syndromes with brain malformations like Marden-Walker syndrome.^16,54,55^ The underlying mechanisms by which PIEZO2 mutations cause brain malformations have not been elucidated, but the early expression of *Piezo2* in the peripheral nervous system could influence the development of mechanoreceptors and their function. Therefore, it remains to be seen whether patients with bi-allelic *PIEZO2* gene loss of function exhibit normal sensory neuron end-terminal morphologies. This question is particularly relevant considering recent evidence that sensory Schwann cells actively participate in the transduction of fine touch stimuli by rapidly-adapting mechanoreceptors.^20,56^ Furthermore, loss-of-function alleles in ion channels like Na_v_1.7 that underlie sensory deficits, like congenital insensitivity to pain, were shown to be associated with aberrant end-terminal morphologies in humans.^57^ The results of our study also raise the important question of whether PIEZO2 gain-of-function mutations in humans may be associated with enhanced pain sensitivity. In most studies looking at patients with pathogenic *PIEZO2* gain-of-function mutations sensory testing was not carried out to quantify pain sensitivity.^15,16,54,58-61^ However, in two studies it was noted that single patients suffered from muscular skeletal pain.^58,60^ Nevertheless since pain has a high prevalence in the healthy population^62^ and these patients have many complicating symptoms, it is presently not possible to draw any strong conclusions. However, our study might warrant re-examining selected patients with quantitative sensory testing.

This profound nociceptor hyper-excitability found in our *Piezo2* knock-in mice strongly resembles physiological sensitization processes that follow strong chemical or mechanical activation of nociceptors in humans, primates and rodents.^36,50,63-66^ The observed changes in mechanosensitive currents measured from recombinant ion channels or isolated nociceptors were remarkably predictive of changes in the *in vivo* sensitivity of nociceptors. For example, R2756 mutations dramatically slowed the closing of PIEZO2 channels a phenomenon that was reflected in ongoing activity of nociceptors after the cessation of the mechanical stimulus. We also show that R2756 mutations strongly influence the excitability of C-fibre nociceptors so that spontaneous activity was seen both *in vitro* and *ex vivo*. Even in wild-type mice mechanical stimuli may enhance nociceptor excitability a phenomenon that was dramatically enhanced in animals with mutant PIEZO2 channels (Fig. 6). This was a very surprising finding as it shows for the first time that it is not only voltage-gated sodium channels like Na_v_1.7, Na_v_1.8 or Na_v_1.9 that have the potential to control nociceptor excitability,^57,67^ but also mechanosensitive channels that are controlled by membrane voltage. In a recent study, a mouse model was generated carrying a Na_v_1.7 (I228M) gain-of-function variant which has been associated with painful small fibre neuropathy in patients.^35^ The Na_v_1.7 (I228M) mice show sensory neuron hyperexcitability to a remarkably similar degree as the mouse mutants we described here; however, in contrast, these mice do not exhibit severe mechanical hypersensitivity as we have shown here for *Piezo2^R2756H/R2756H^* and *Piezo2^R2756K/R2756K^* mice (Fig. 8).

It is even conceivable that *in vivo* some PIEZO2 channels can be directly opened by membrane voltage even in the absence of mechanical stimuli, a phenomenon that we have demonstrated for recombinantly expressed *Piezo1* channels.^12^ Pure voltage-gating of the PIEZO1 channel was seen when the channel was mutated or was stimulated with the chemical agonist Yoda.^12^ Both PIEZO1 and PIEZO2 can be sensitized or modulated by other proteins including stomatin like protein-3 (STOML3) and MyoD (myoblast determination)–family inhibitor proteins (MDFIC and MDFI).^17,68^ Such modulators could plausibly push PIEZO2 into a hyperexcitable state that may promote voltage-gating. Thus, other mechanisms not involving missense mutations could promote PIEZO2-dependent nociceptor sensitization. Activation of nociceptors by inflammatory mediators or algogens, like capsaicin,^5^ will strongly depolarize sensory endings thus potentially relieving voltage block of PIEZO2 channels. Membrane depolarization, which in the very compact nociceptor ending may be considerable, has the potential to mimic relief of voltage block that keeps the threshold to activate nociceptors high. Thus, we propose that voltage control of abundant PIEZO2 channels in most nociceptors is a major final mediator of nociceptor sensitization caused by strong nociceptive stimuli. Nociceptor sensitization is central to many chronic pain states making pharmacological manipulation of PIEZO2 voltage sensitivity an attractive target for pain therapy.

## Supplementary Material

awae227_Supplementary_Data

## Data Availability

Data are available from the corresponding author upon reasonable request.
