# Supplementary material for: Piezo2 voltage-block regulates mechanical pain sensitivity
Source: Brain. 2024 Jul 10;147(10):3487–500. doi: 10.1093/brain/awae227 (PMC11449130; doi:10.1093/brain/awae227)
Supplement: awae227_Supplementary_Data [file awae227_supplementary_data.pdf]

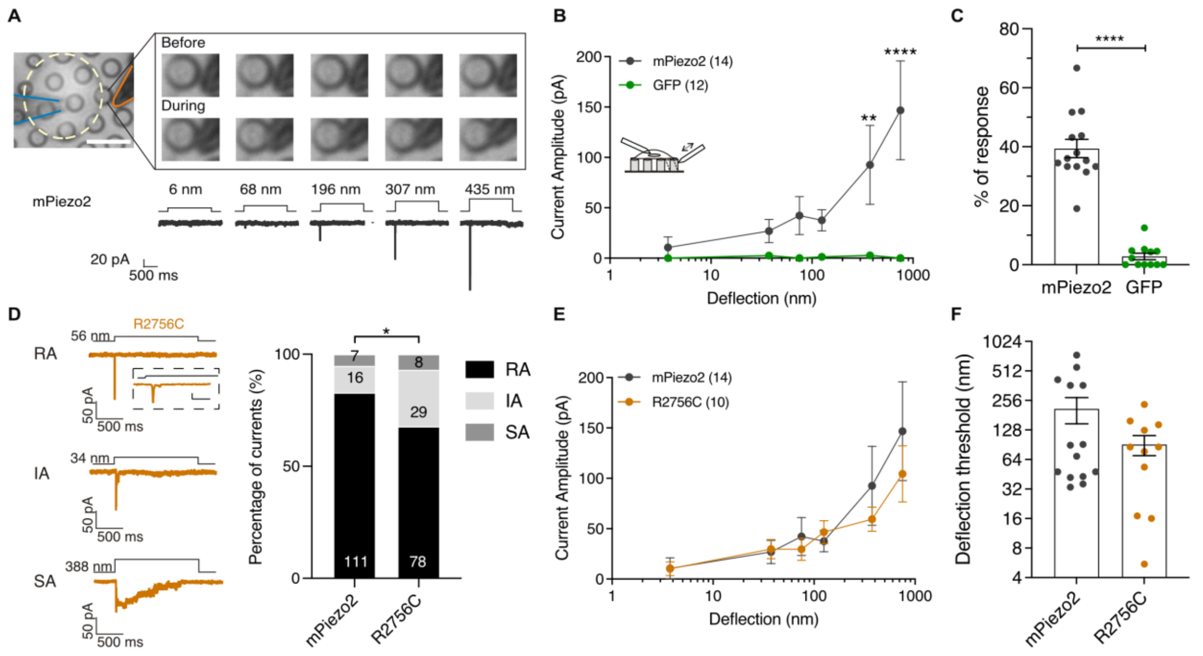

Supplementary Figure 1. R2756C variant displayed less RA deflection-gated currents compared to control in N2a$^{Piezo1-/-}$. (**A**) Bright-field picture of a N2a$^{Piezo1-/-}$ cell overexpressing mPiezo2 and cultured on pillar arrays. The insert shows an amplification of the stimulated pilus and the sequential stimuli applied with their corresponding deflection-gated inward current (grey traces). Traces correspond to RA-currents. Note that the larger the deflection, the larger the MA current. (**B**) Stimulus-response plot of the deflection sensitive currents from N2a$^{Piezo1-/-}$ overexpressing mPiezo2 (grey) and GFP (green) (Two-way ANOVA, Sidak's multiple comparison test; ***P*=0.002, *****P*<0.0001). (**C**) Percentage of response to deflection stimuli (39.39 ± 3.08 and 2.8 ± 1.09 % for mPiezo2- and GFP-transfected N2a$^{Piezo1-/-}$ cells, respectively). The total amount of stimuli was considered as 100%. Each dot represents the percentage of individual cells (Student's t test; *****P*<0.0001). (**D**) *Left*, Representative traces of the three types of deflection-gated currents from N2a$^{Piezo1-/-}$ cells overexpressing R2765C mutant. Rapidly adapting (RA), intermediate adapting (IA) and slowly adapting (SA) currents are shown. *Right*, Proportion of RA-currents is decreased in R2756C variant. Numbers in the histograms show the number of currents recorded (χ^2 test, **P*=0.01). (**E**) Deflection-current amplitude relationship of mPiezo2 and R2756C mutant. (**F**) Histogram showing that mPiezo2 and R2756C variant showed similar deflection thresholds. Data was plotted as mean ± s.e.m.

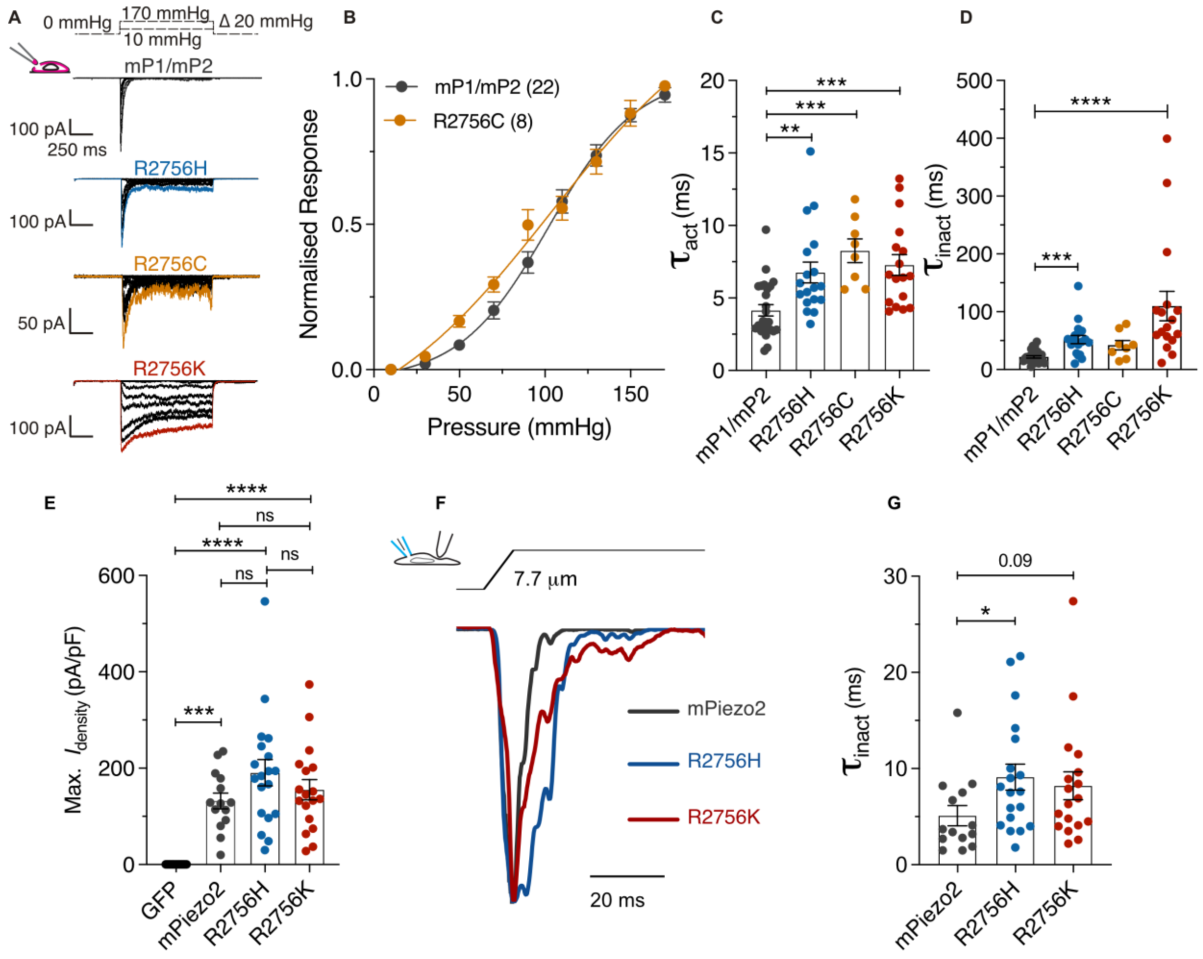

Supplementary Figure 2. Piezo2 variants displayed altered inactivation kinetics. (**A**) Representative recordings of stretch-sensitive currents of outside-out patches clamped at -60 mV from N2a$^{Piezo1-/-}$ cells overexpressing the chimeric channel mP1/mP2 and mutants. The dashed lines above show the pressure protocol applied. (**B**) Stretch-response curves and mechanical sensitivity of mP1/mP2 and R2756C chimeric channels. The peak currents were normalised according to the maximum amplitude current recorded. (**C-D**) Time constant of activation (τ_{act}, **C**), and inactivation (τ_{inact}, **D**) change in mutants of the chimeric channels. The values correspond to the currents recorded at 130 mmHg pulse (Kruskal-Wallis test, **P*=0.01; ****P*<0.001, *****P*<0.0001). (**E**) Max. current density (Max. $I_{density}$; pA/pF) plot of Piezo2-indentation gated currents overexpressed in N2a$^{Piezo1-/-}$ cells showing no differences between wild type and mutant channels (Kruskal-Wallis test, ****P*=0.0004, *****P*<0.0001). (**F, G**) R2756H variant showed slower inactivation kinetics compared to wild type channels. Each dot represents the inactivation kinetics values of mechanosensitive currents evoked at 7 µm (Kruskal-Wallis test, **P*=0.02). In all cases, values were plotted as mean ± s.e.m.

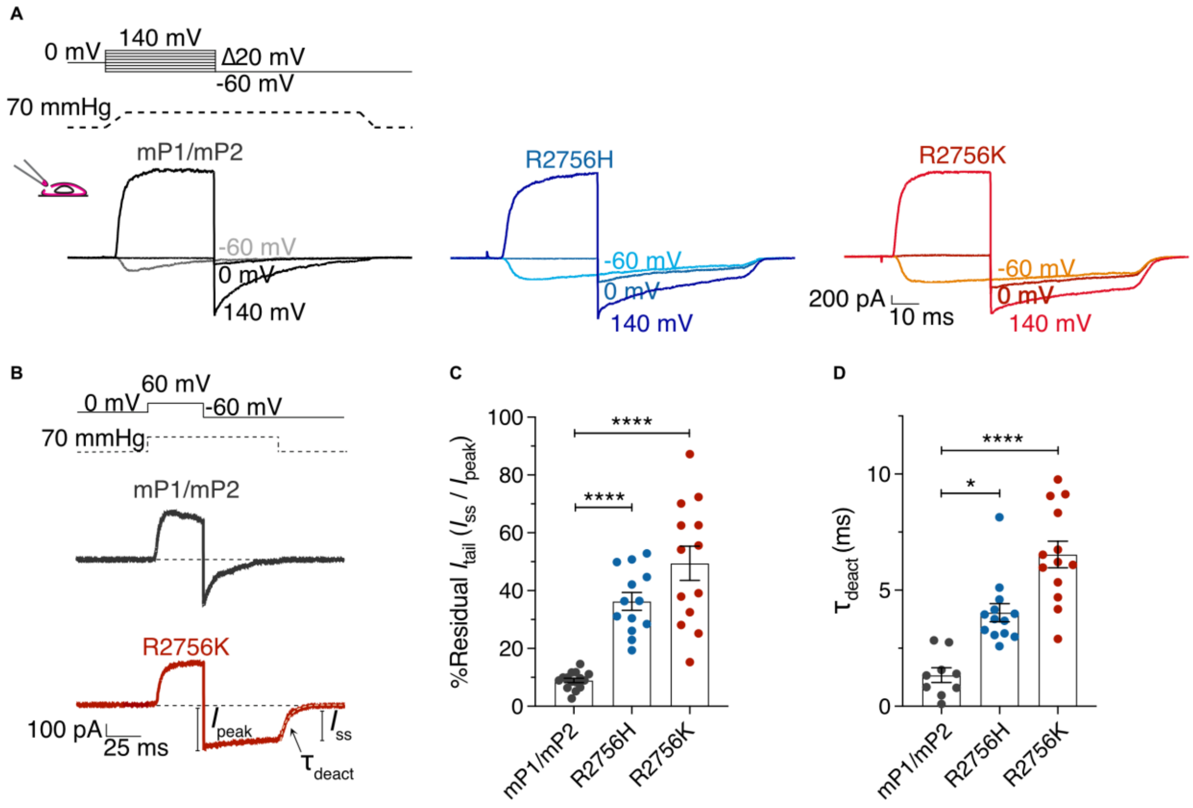

Supplementary Fig. 3. Channel availability and deactivation change in the stretch-sensitive mutants of mP1/mP2 channels. (**A**) Example traces of the tail current protocol performed in N2a$^{Piezo1\text{-/-}}$ cells overexpressing the chimeric variants in presence of 70 mmHg of pressure. Note that the tail currents in the mutants are larger at -60 and 0 mV compared to the control, indicating increased channel availability. (**B**) Representative traces from outside-out patches clamped to a voltage step of +60 mV followed by a step of -60 mV in the presence of pressure stimuli of 70 mmHg. Peak (I_{peak}) and steady-state (I_{ss}) currents are indicated. (**C**) Ratio of I_{peak} to I_{ss} from the instantaneous tail currents (I_{tail}) at -60 mV (Dunnett test; ****P<0.0001). (**D**) Kinetics of the inactivation-deactivation state (τ_{deact}) from mP1/mP2 variants (Kruskal-Wallis test; *P=0.01, ****P<0.0001). Data was plotted as mean ± s.e.m.

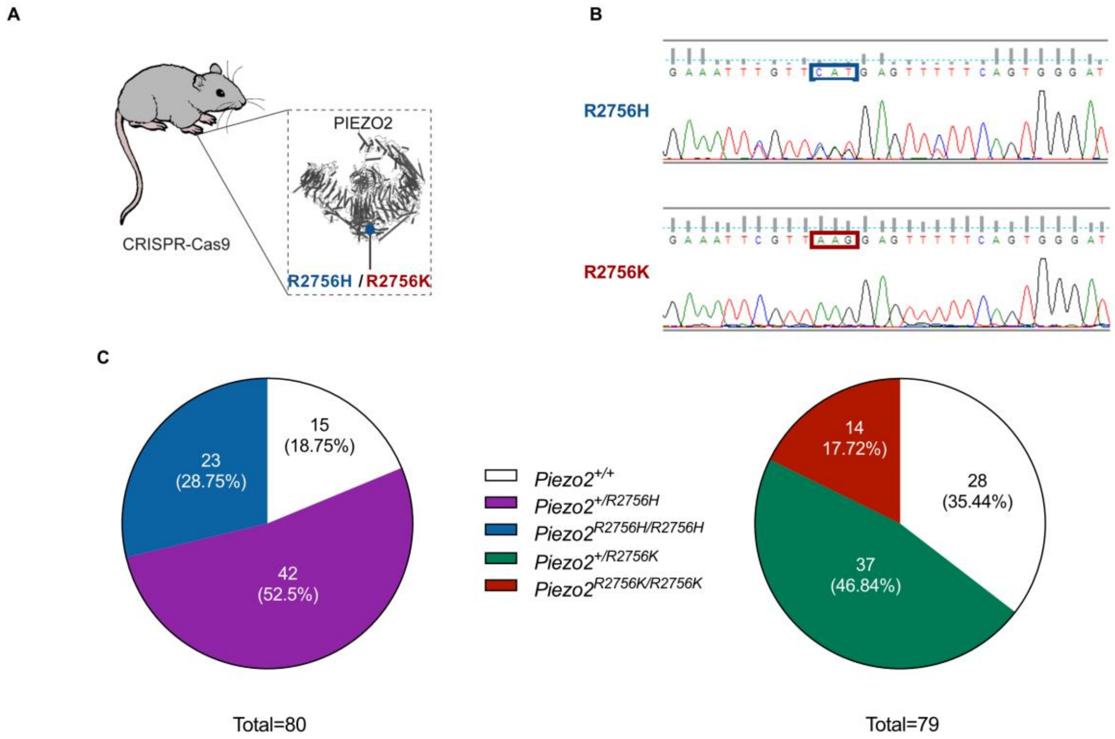

Supplementary Fig. 4. *Piezo2 knock-in* were born with normal Mendelian ratios. (**A**) Cartoon indicating the global insertion of mutations R2756H and R2756K in Piezo2. (**B**) Sequencing results from founder mice carrying the mutations R2756H (*above*) and R2756K (*below*) in Piezo2. The substituted nucleotides are highlighted inside the squares. Note that in *Piezo2* wild type animals Arginine is encoded by CGT codon (*not shown*). (**C**) Proportion of mice from *Piezo2 knock-in* mutants. Numbers show the total number of animals. For *Piezo2*$^{R2756H/R2756H}$ and *Piezo2*$^{R2756K/R2756K}$ 10 and 12 litters were considered, respectively (χ^2 test; $P>0.05$).

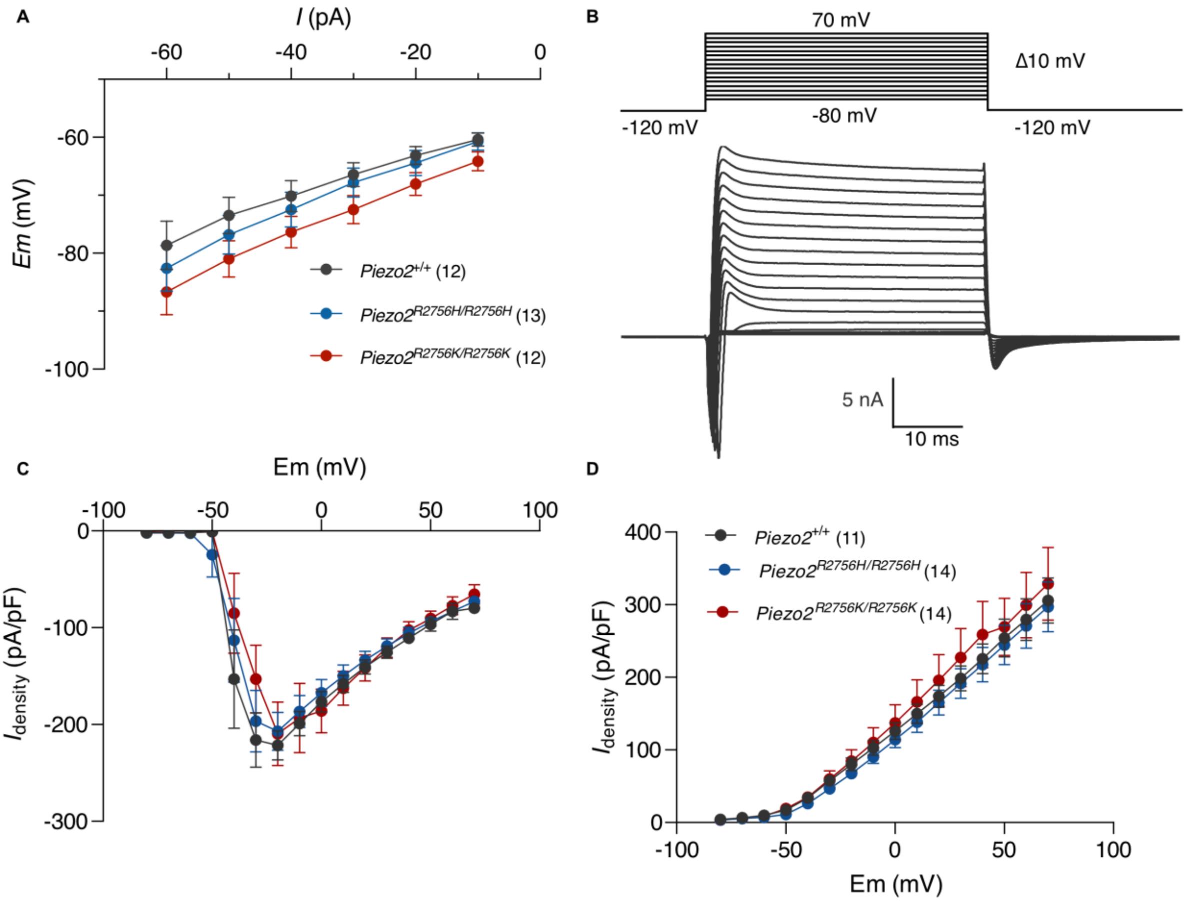

Supplementary Fig. 5. Macroscopic inward and outward currents do not change in mechanoreceptors from *Piezo2* mutants. (**A**) Current (*I*) -voltage (Em) plot showing that input resistance did not change in mechanoreceptors from *Piezo2 knock-in* mice. (**B**) Representative traces from inward and outward currents recorded from a wild type mechanoreceptor. (**C-D**) Voltage (Em) - Current density ($I_{density}$) relationships showing that inward currents (**C**) and outward currents (**D**) from mechanoreceptors were similar in *Piezo2*$^{+/+}$ and mutants. Data was plotted as mean ± s.e.m.

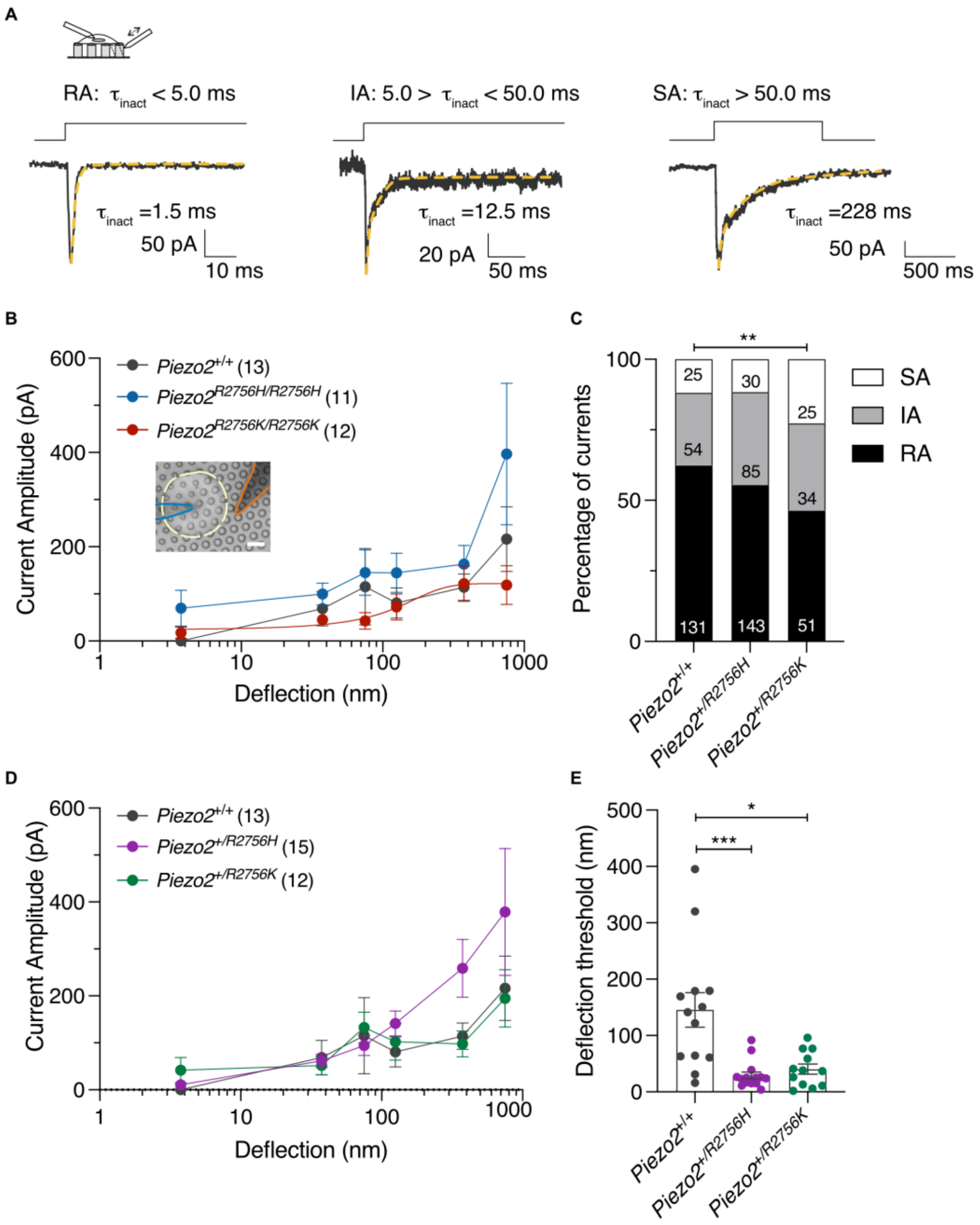

Supplementary Fig 6. Heterozygous conditions of *Piezo2 knock-in* mice showed more sensitive deflection-gated currents in mechanoreceptors. (**A**) Representative traces of the three types of MA currents from isolated sensory neurons recorded using pillar arrays. Dashed line indicates τ_{inact} fit. (**B**) Deflection-current amplitude relationship of mechanoreceptors from *knock-in* and control mice. Note that neurones from *Piezo2*$^{R2756K/R2756K}$ reached current saturation, indicating increased sensitivity to mechanical stimuli. (**C**) Histograms showing that *Piezo2*$^{+/R2756K}$ mechanoreceptors evoked less RA currents compared to wild type cells (χ^2 test, ***P*=0.009). Numbers indicate the total of currents recorded. (**D**) Deflection-current amplitude plot showing no differences between wild type mechanoreceptors and neurones from both heterozygous mice. (**E**) Deflection thresholds were lower in mechanoreceptors from both heterozygous mice compared to wild type cells. (Kruskal-Wallis test, **P*=0.01, ****P*=0.0004) Data was plotted as mean ± s.e.m.

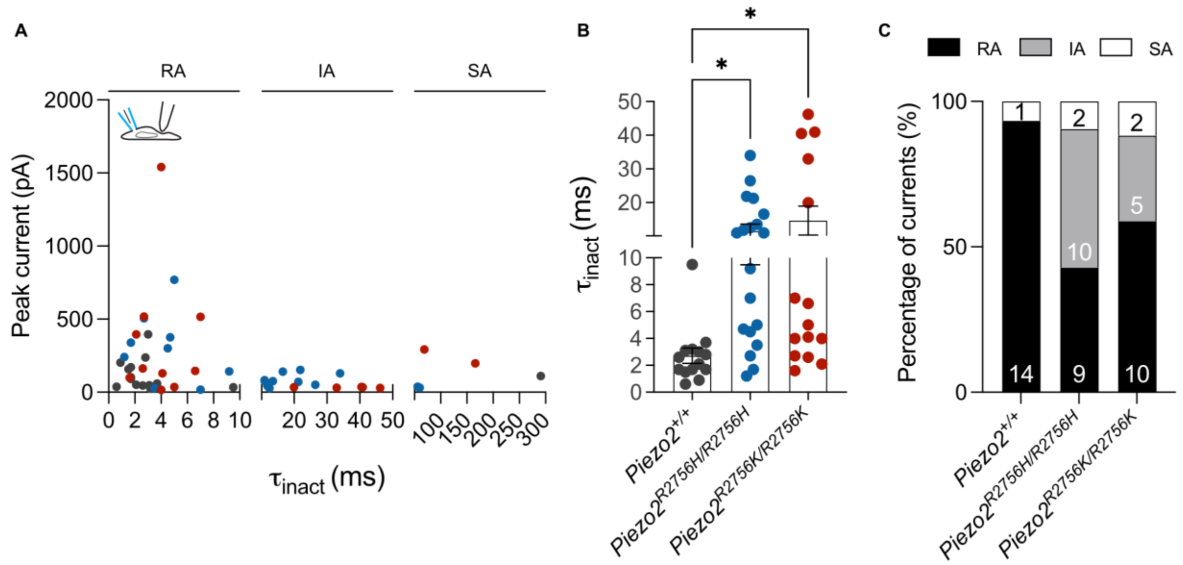

Supplementary Figure 7. Mechanoreceptors from Piezo2 mutants showed slower inactivation kinetics. (**A**) τ_{inact} – Peak current relationship showing that neurones from Piezo2 mutants showed a higher proportion of currents with slower inactivation kinetics compared to wild type cells. Each dot represents the inactivation kinetics value from the last stimulus applied in each cell. (**B**) Inactivation kinetics (τ_{inact}) plot from RA and IA currents in mechanoreceptors from mutant and wild type neurones. (Kruskal-Wallis test, *P<0.05). (**C**) Stacked histogram indicating a reduction of RA currents proportion in Piezo2 mutants compared to wild type.

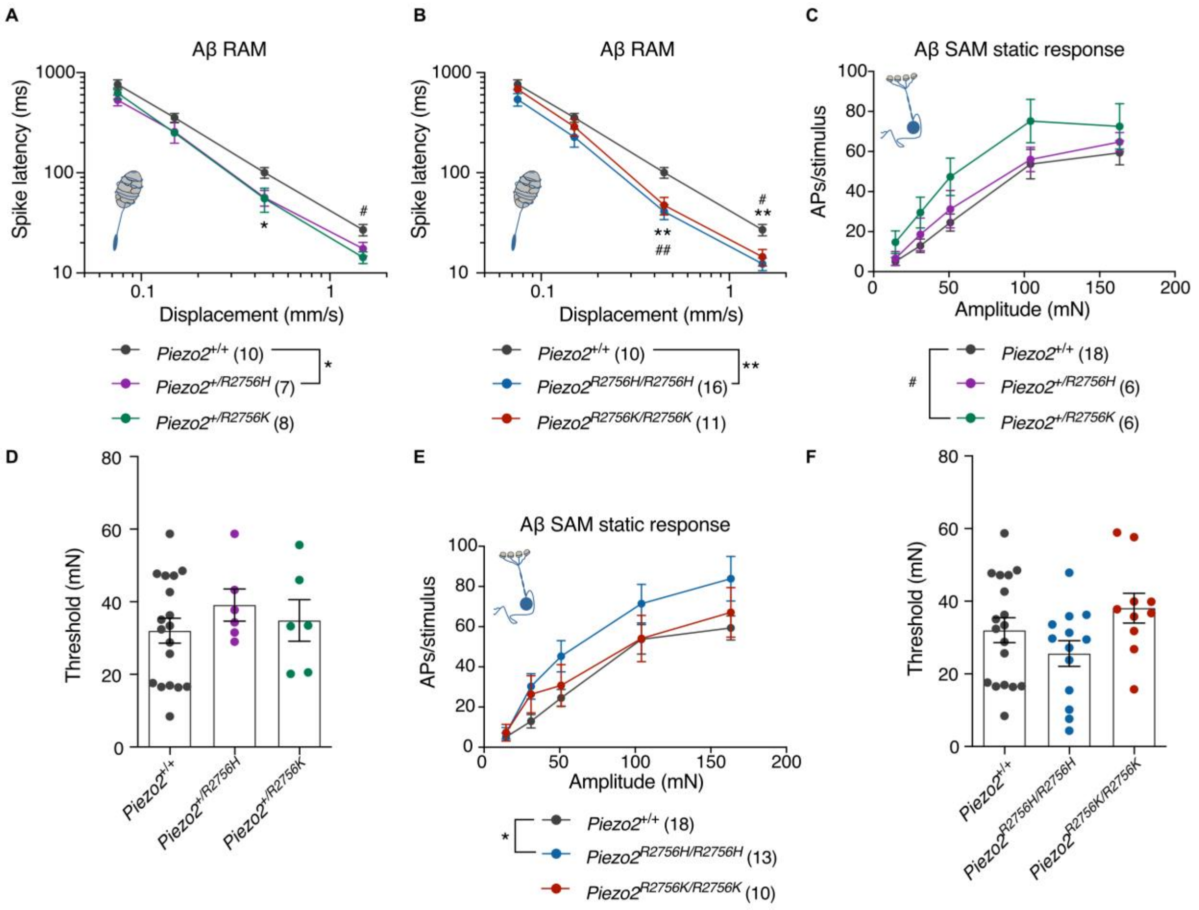

Supplementary Fig. 8. Mechanical thresholds from Aβ SAM fibres were not affected in *Piezo2 knock-in* mice. (**A-B**) Rapidly adapting afferents from *Piezo2 knock-in* mice displayed decreased AP firing latencies at velocity stimuli indicating increased sensitivity to mechanical stimuli (Two-way ANOVA with Sídák post hoc analysis; (**A**), *P<0.05, $^{\#}P$=0.01; (**B**) **P<0.01, $^{\#}P$=0.03, $^{\#\#}P$=0.009). (**C-D**) Slowly adapting fibres showed a slightly AP firing increase in *Piezo2*$^{+/R2756K}$ mutants (Two-way ANOVA with Sídák post hoc analysis, $^{\#}P$=0.01), but mechanical thresholds were not impaired compared to wild type. (**E-F**) SAM afferents from *Piezo2*$^{R2756H/R2756H}$ displayed higher AP firing compared to wild type fibres during sustained mechanical stimuli (Two-way ANOVA with Sídák post hoc analysis, *P=0.01), but no differences in mechanical thresholds were observed.

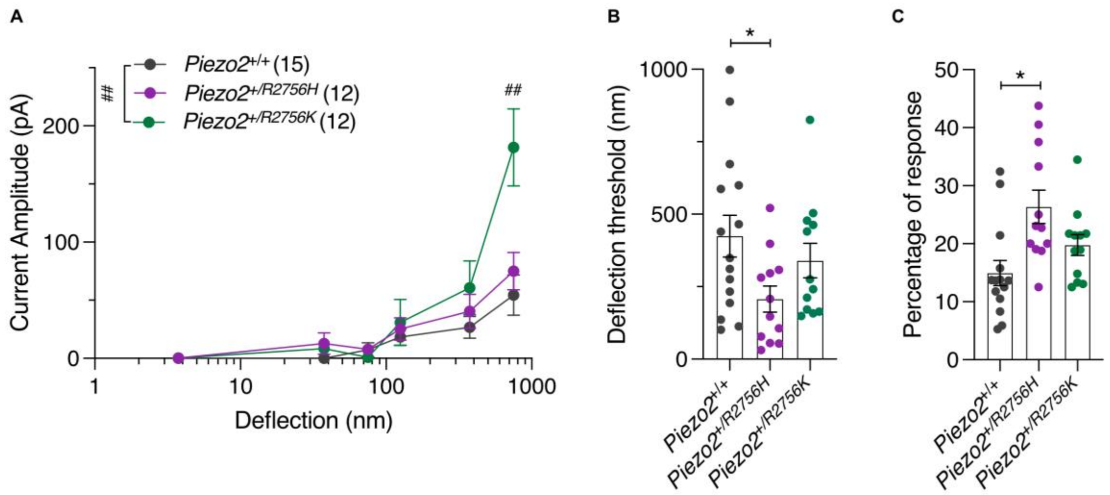

Supplementary Fig. 9. *Piezo2*$^{+/R2756H}$ nociceptors displayed more sensitive deflection-gated currents than controls. (**A**) Deflection-current amplitude relationships of nociceptors from heterozygous *Piezo2 knock-in* and control mice (Mann Whitney test $^{\#\#}P$=0.003; additionally an ordinary Two-way ANOVA indicated differences between *Piezo2*$^{+/+}$ and *Piezo2*$^{+/R2756K}$, $^{\#\#}P$=0.003). (**B**) Plots showing that deflection thresholds were lower in *Piezo2*$^{+/R2756H}$ neurones compared to wild type (One-Way ANOVA, *P=0.03). (**C**) Dot plot showing that *Piezo2*$^{+/R2756H}$ cells showed enhanced responsiveness to deflection stimuli compared to controls (One-Way ANOVA, *P=0.03). Data was plotted as mean ± s.e.m.

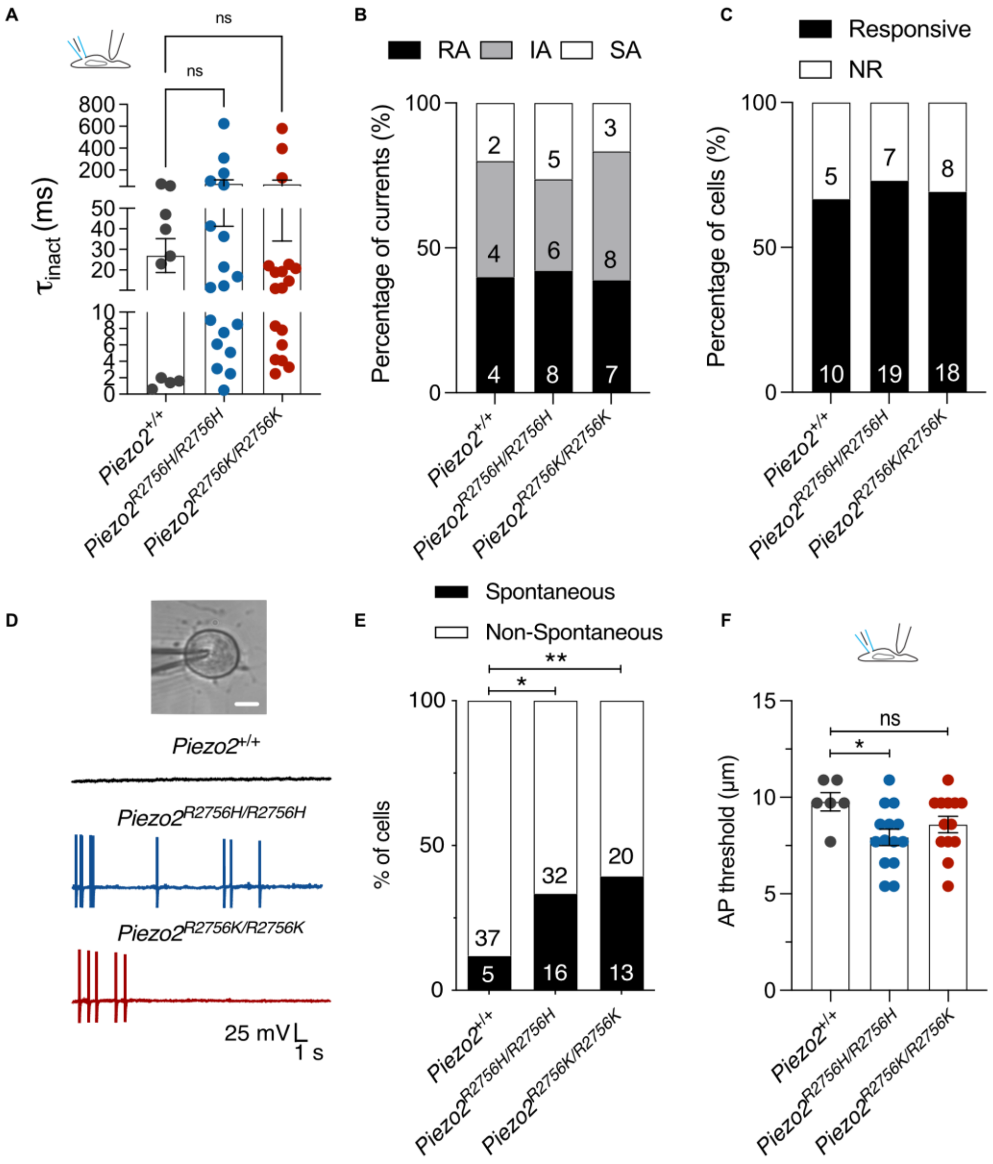

Supplementary Fig. 10. *Piezo2 knock-in* nociceptors showed spontaneous AP firing in absence of mechanical stimuli. (**A**) Inactivation kinetics plot from nociceptors in *Piezo2* mutants. (**B**) Stacked histogram indicating similarities in the proportion of mechanosensitive currents proportion in Piezo2 compared to wild type. (**C**) Histogram showing that proportion of responsive neurones to mechanical stimuli in the mutants is similar to control cells. (**D-E**) Representative traces of current clamp recordings and percentage of neurones showing spontaneous activity in nociceptors from control and *knock-in* mice. Numbers indicate number of cells recorded (χ^2 test, **P*=0.01, ***P*=0.005). (**F**) Histogram indicating that isolated nociceptors from *Piezo2*$^{R2756H/R2756H}$ displayed a lower mechanical threshold to induce AP firing when performing current clamp recordings (Kruskal-Wallis test, **P*=0.03).

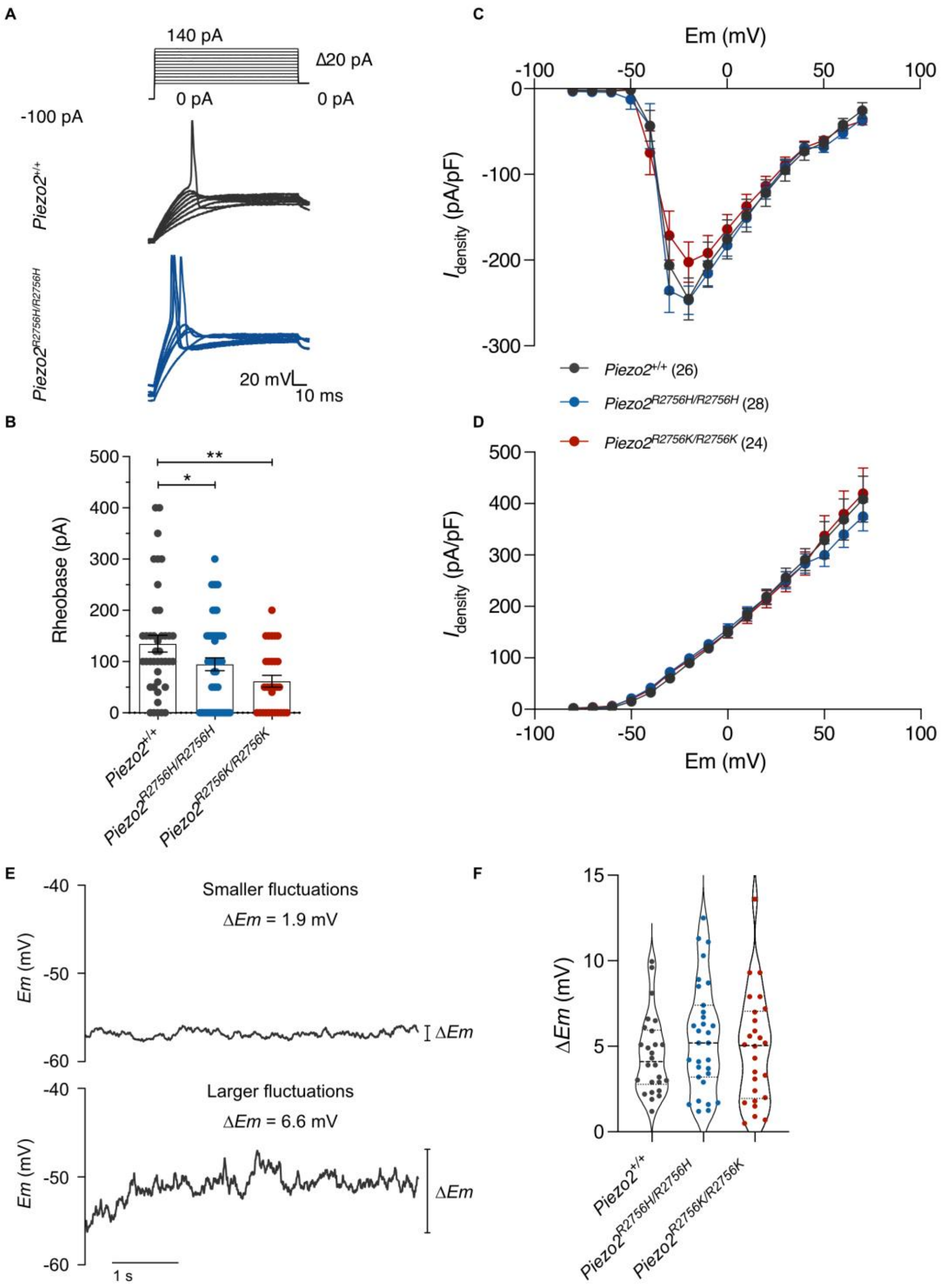

Supplementary Fig. 11. Nociceptors from *Piezo2 knock-in* mice displayed hyperexcitability. (**A**) Representative traces from APs evoked at different current step injections in nociceptors from *Piezo2*$^{+/+}$ and *Piezo2*$^{R2756H/R2756H}$. Note that *Piezo2*$^{R2756H/R2756H}$ cells showed lower thresholds. (**B**) Dot plots showing that *Piezo2 knock-in* neurones exhibited lower AP threshold firings (Rheobase) (One-way ANOVA, **P*=0.03; ***P*=0.001). (**C-D**) Voltage (Em)-Current density (I_{density}) relationships showing that inward currents (**C**) and outward currents (**D**) from nociceptors were similar in *Piezo2*$^{+/+}$ and mutants. Data was plotted as mean ± s.e.m. (**E**) Example traces of current clamp recordings from nociceptors showing smaller and larger spontaneous voltage fluctuations. (**D**) Violin plot showin that the ΔEm of the spontaneous fluctuations was not different between wild type and mutant cells.

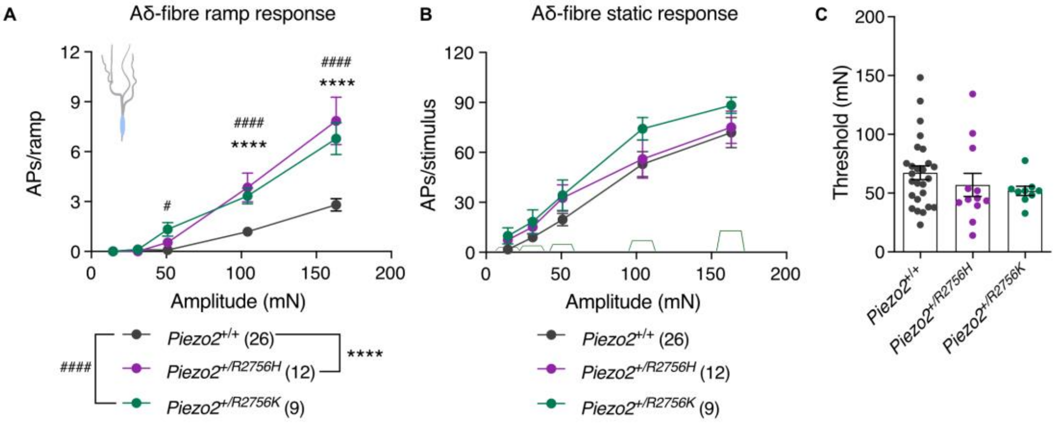

Supplementary Fig. 12. Aδ-nociceptor firing is enhanced in heterozygous *Piezo2 knock-in* mice during the dynamic phase. (**A**) Aδ-fibre firing activity during ramp phase (Two-way ANOVA with Sídák post hoc analysis, $^{\#}P$<0.05, ****P<0.0001, $^{\#\#\#\#}P$<0.0001). Note that heterozygous mice showed enhanced firing compared to controls. (**B**) Static phase responses from Aδ-fibres in *Piezo2*$^{+/+}$ and heterozygous animals. No differences were found in the static phase of the mechanical stimuli. (**C**) Dot plot showing that Aδ-nociceptors from heterozygous *Piezo2 knock-in* mice responded similarly to mechanical stimuli compared to controls. Data are presented as mean ± s.e.m.

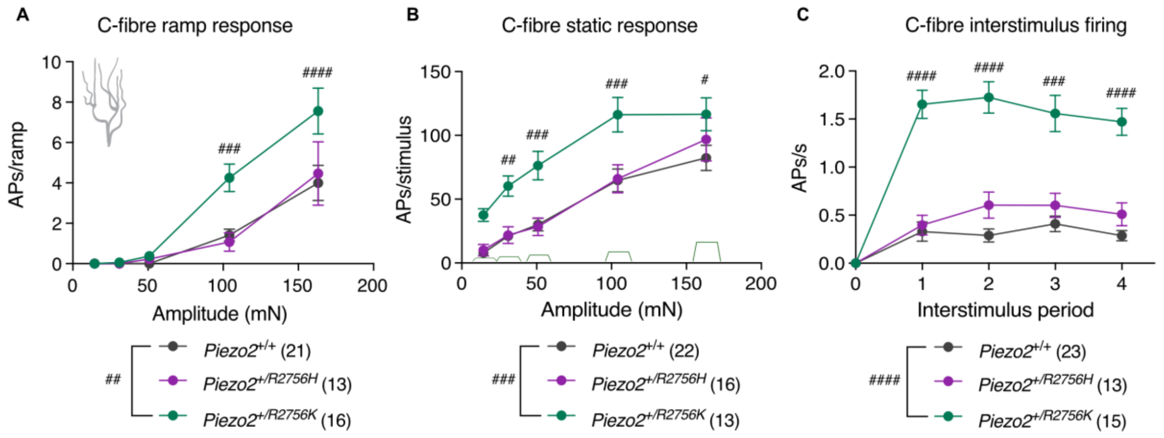

Supplementary Fig. 13. *Piezo2*$^{+/R2756K}$ nociceptors exhibited after discharge firing activity. (**A-C**) AP firing activity of C-fibres during ramp phase (**A**), Two-way ANOVA with Sídák post hoc analysis, $^{##}P$=0.001, $^{###}P$=0.0004, $^{####}P$<0.0001), static phase (**B**), Two-way ANOVA with Sídák post hoc analysis, $^{#}P$=0.02, $^{##}P$=0.006, $^{###}P$<0.001) and interstimulus firing (**C**), Two-way ANOVA with Sídák post hoc analysis, $^{###}P$=0.0001, $^{####}P$<0.0001). Data are presented as mean ± s.e.m.

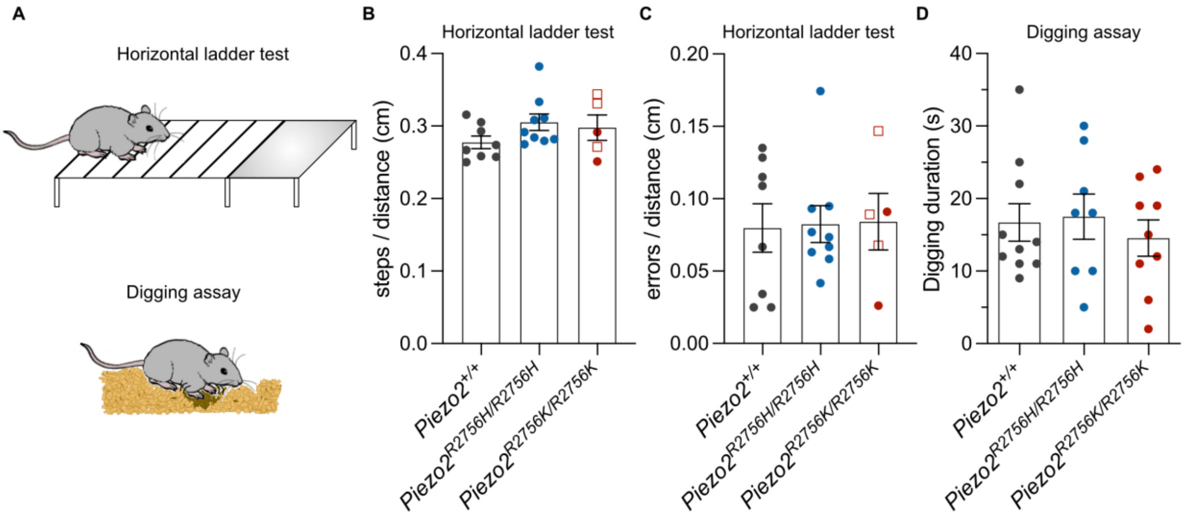

Supplementary Fig. 14. *Piezo2* mutants do not develop proprioceptive deficits nor spontaneous pain behaviour. (**A**) Cartoons representing the horizontal ladder and digging assays. (**B-C**) Histograms showing that *Piezo2 knock-in* mice did not displayed differences when performing horizontal ladder crossing walks compared to wild type animals. (**D**) Digging responses were measured as an indication of spontaneous pain (well-being). Both, *Piezo2*$^{R2756H/R2756H}$ and *Piezo2*$^{R2756K/R2756K}$ animals showed similar digging responses. Empty squares indicate *post-mortem* examined *Piezo2*$^{R2756K/R2756K}$ mice that developed scoliosis.

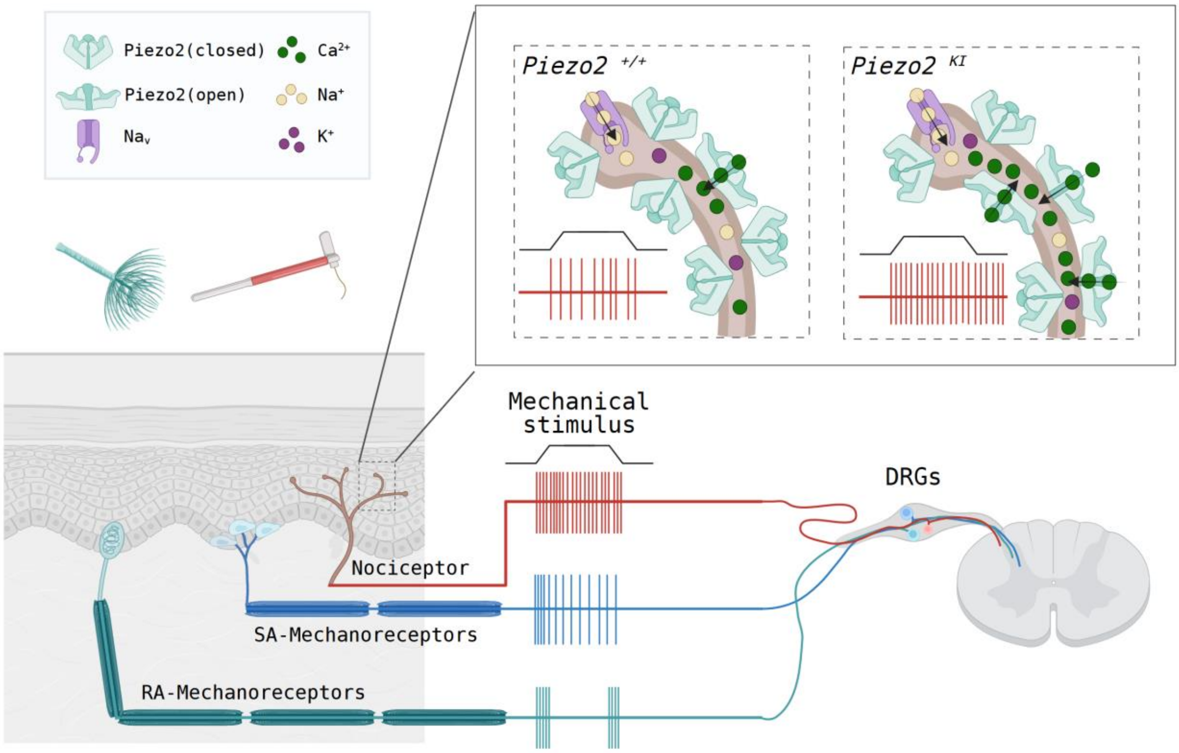

Supplementary Fig. 15. Graphical Abstract. Model of how Piezo2 point mutations lead to nociceptor hyper excitability. The figure was generated using Biorender.

Supplementary Table 1. Biophysical properties of N2a$^{Piezo1-/-}$ cells overexpressing mPiezo2 mutants

	**mPiezo2**	**R2756H**	**R2756C**	**R2756K**
**Cells (no. of currents)**	14 (134)	11 (130)	10 (115)	11 (172)
**Latency (ms)**	**1.14 ± 0.62**	**2.47 ± 0.37****	**2.54 ± 0.37****	**2.34 ± 0.27**
**RA**	1.2 ± 0.83	2.44 ± 0.55	2.07 ± 0.35	2.32 ± 0.35
**IA**	1.35 ± 0.28	2.47 ± 0.54	3.34 ± 0.65	2.61 ± 0.76
**SA**	1.29 ± 0.41	3.2 ± 0.65	7.76 ± 3.9	2.67 ± 0.81
τ_{act} **(ms)**	**0.65 ± 0.04**	**0.86 ± 0.05****	**0.68 ± 0.04**	**0.74 ± 0.06**
**RA**	0.60 ± 0.05	0.72 ± 0.05	0.58 ± 0.03	0.62 ± 0.04
**IA**	1.06 ± 0.17	1.22 ± 0.13	0.97 ± 0.19	1.11 ± 0.27
**SA**	1.14 ± 0.23	0.81 ± 0.18	0.9 ± 0.25	0.96 ± 0.16
τ_{inact} **(ms)**	**14.02 ± 5.64**	**21.08 ± 5.86*****	**18.49 ± 6.35**	**16.51 ± 3.27**
**RA**	0.83 ± 0.08	1.76 ± 0.1*	1.04 ± 0.12	0.99 ± 0.1
**IA**	17.62 ± 3.28	16.74 ± 2.01	15.25 ± 1.75	17.99 ± 3.07
**SA**	212.2 ± 75.35	198.2 ± 50.76	170.7 ± 56.35	114.4 ± 17.02

Number of cells and currents analysed from cells overexpressing pathogenic mutations of Piezo2. All data come from, at least, three different transfections. Mechanical latency, τ_{act} and τ_{inact} are shown for all cells including the values or RA, IA and SA groups. (Kruskal-Wallis test, *P=0.04, **P<0.01,***P<0.001). Values are shown as mean ± s.e.m.

Supplementary Table 2. Biophysical properties of indentation-gated currents from Piezo2 variants in N2a$^{Piezo1-/-}$ cells

	Piezo2	R2756H	RH2756K
**Cells**	14	19	18
**Max. $I_{density}$ (pA/pF)**	131.8 ± 16.4	190.5 ± 27.1	155.1 ± 21.0
τ_{act} **(ms)**	3.1 ± 0.2	2.7 ± 0.2	3.4 ± 0.3
τ_{inact} **(ms)**	5.1 ± 1.0	**9.1 ± 1.3***	8.2 ± 1.4
**Mechanical threshold (µm)**	3.9 ± 0.3	**2.9 ± 0.1***	**2.9 ± 0.1***

Number of cells analysed from cells overexpressing pathogenic mutations of Piezo2. All data come from more than three different transfections. (Kruskal-Wallis test, *P<0.05). Values are shown as mean ± s.e.m. τ_{act} *and* τ_{inact} values are taken from currents evoked with a 7.7µm stimulus.

Supplementary Table 3. Biophysical properties of deflection gated currents in mechanoreceptors from *Piezo2*^{R2756H} and *Piezo2*^{R2756K} mice

	Piezo2$^{+/+}$	*Piezo2*$^{+/R2756H}$	*Piezo2*$^{R2756H/R2756H}$	*Piezo2*$^{+/R2756K}$	*Piezo2*$^{R2756K/R2756K}$
**Animals (m,f)**	6 (5,1)	7 (6,1)	6 (4,2)	4 (3,1)	5 (3,2)
**Cells (no. of currents)**	13 (210)	15 (258)	11 (145)	12 (110)	12 (123)
**Cell size (µm)**	37.96 ± 0.98	41.33 ± 0.69	37.3 ± 1.05	39.44 ± 1.96	36.87 ± 0.68
**HP (ms)**	0.66 ± 0.05	0.65 ± 0.12	0.6 ± 0.11	0.7 ± 0.08	0.61 ± 0.08
**Em_{rep} (mV)**	-66.72 ± 1.04	-66.78 ± 1.64	-65.16 ± 2.33	-64.89 ± 1.08	-63.13 ± 1.31
**Latency (ms)**	**3.19 ± 0.42**	**2.77 ± 0.34**	**4.98 ± 0.64**	**5.23 ± 0.78******	**3.86 ± 0.45******
**RA**	3.98 ± 0.9	1.73 ± 0.3	4.79 ± 0.87	4.73 ± 1.07**	3.21 ±0.53*
**IA**	3.10 ± 0.87	4.49 ± 0.86	4.64 ± 1.18	5.05 ± 1.39	5.57 ± 1.25**
**SA**	2.22 ± 0.81	2.01 ± 0.47	5.92 ± 1.81	5.52 ± 1.62	4.18 ± 0.91
**τ_{act} (ms)**	**0.87 ± 0.04**	**1.2 ± 0.06****	**1.43 ± 0.09******	**1.52 ± 0.09******	**1.33 ± 0.11*****
**RA**	0.71 ± 0.04	0.84 ± 0.05	1.27 ± 0.13****	1.25 ± 0.11****	0.97 ± 0.07**
**IA**	1.07 ± 0.09	1.72 ± 0.15**	1.57 ± 0.19	1.65 ± 0.15**	1.96 ± 0.35*
**SA**	1.26 ± 0.12	1.46 ± 0.21	1.62 ± 0.23	1.85 ± 0.26	1.96 ± 0.32
**τ_{inact} (ms)**	**23.86 ± 3.83**	**31.43 ± 6.05**	**42.37 ± 8.96****	**61.89 ± 14.67****	**32.2 ± 8.98**
**RA**	1.79 ± 0.1	1.82 ± 0.11	2.43 ± 0.16**	1.95 ± 0.14	1.94 ± 0.14
**IA**	18.8 ± 1.57	16.16 ± 1.32	14.02 ± 1.36*	17.35 ± 2.16	13.48 ± 1.75
**SA**	149.5 ± 16.8	215.9 ± 37.45	184.4 ± 34.45	246.4 ± 50.27	181.4 ± 45.45

Sex and number of animals (m, male, f, female) used in the study. Cells and currents analysed in mechanoreceptors for each genotype are shown. Cell size and half peak (HP) of APs were used to classified DRG neurones into mechanoreceptors. Mechanical latency, τ_{act} and τ_{inact} are shown for all genotypes including the values or RA, IA and SA groups. (Kruskal-Wallis test, *P<0.05, **P<0.01, ***P<0.001, ****P<0.0001). Values are shown as mean ± s.e.m.

Supplementary Table 4. Biophysical properties of indentation-gated currents on mechanoreceptors from *Piezo2*^{R2756H} and *Piezo2*^{R2756K} mice

	Piezo2$^{+/+}$	*Piezo2*$^{R2756H/R2756H}$	*Piezo2*$^{R2756K/R2756K}$
**Animals**	4	3	3
**Cells**	16	24	20
**Cell size (µm)**	37.1 ± 1.3	35.5 ± 0.6	36.6 ± 1.2
**HP (ms)**	0.6 ± 0.05	0.7 ± 0.08	0.8 ± 0.1
**Em_{rep} (mV)**	-63.4 ± 1.7	-62.7 ± 0.5	**-57.6 ± 1.5****
**Mechanical threshold (µm)**	7.5 ± 0.5	6.6 ± 0.4	7.2 ± 0.4
**I_{max} (pA)**	117.9 ± 26.1	173.3 ± 41.7	247.5 ± 90.4
**τ_{act} (ms)**	3.8 ± 0.8	5.1 ± 0.6	4.5 ± 0.8
**τ_{inact} (ms)**	2.7 ± 0.5	**11.5 ± 2.0***	**14.6 ± 4.3***
***RA* τ_{inact} (ms)**	2.7 ± 0.5	4.3 ± 0.8	3.9 ± 0.5

Number of animals and responsive cells analysed in the study. Cell size and half peak (HP) of APs were used to classify DRG neurones into mechanoreceptors. τ_{inact} values from RA and IA currents. (Em_{rep}: Kruskal-Wallis test, ***P*=0.002; τ_{inact}: one-way ANOVA, **P*<0.05). Values are shown as mean ± s.e.m.

Supplementary Table 5. Biophysical properties of deflection gated currents in nociceptors from *Piezo2 knock-in* mice

	Piezo2 $^{+/+}$	***Piezo2*** $^{+/R2756H}$	***Piezo2*** $^{R2756H/R2756H}$	***Piezo2*** $^{+/R2756K}$	***Piezo2*** $^{R2756K/R2756K}$
**Animals (m,f)**	5 (4,1)	4 (3,1)	4 (2,2)	3 (2,1)	4 (3,1)
**Cells (no. of currents)**	15 (77)	12 (62)	13 (97)	12 (54)	14 (113)
**Cell size (μm)**	26.67 ± 1.13	23.61 ± 0.66	26.04 ± 1.32	24.84 ± 0.72	24.45 ± 0.86
**HP (ms)**	2.21 ± 0.27	2.54 ± 0.36	2.59 ± 0.31	1.75 ± 0.17	2.18 ± 0.25
**Em_{rep} (mV)**	-54.6 ± 2.6	-52.5 ± 2.7	-52.9 ± 3.1	-54.7 ± 2.4	-49.7 ± 3.2
**Latency (ms)**	**1.44 ± 0.16**	**2.24 ± 0.45**	**2.41 ± 0.43**	**2.96 ± 0.55*** **	**2.68 ± 0.34** **
**RA**	1.25 ± 0.15	1.85 ± 0.53*	2.31 ± 0.59	2.29 ± 0.49**	2.28 ± 0.44**
**IA**	1.81 ± 0.53	2.2 ± 0.56	2.78 ± 0.76	4.41 ± 1.14	2.73 ± 0.53
**SA**	2.29 ± 0.77	2.95 ± 1.28	2.25 ± 0.66	3.7 ± 1.63	3.96 ± 0.92
τ_{act} **(ms)**	**0.75 ± 0.07**	**0.83 ± 0.05**	**1.00 ± 0.11**	**1.02 ± 0.09** **	**1.24 ± 0.13***
**RA**	0.6 ± 0.05	0.67 ± 0.8	0.63 ± 0.05	0.77 ± 0.09	0.73 ± 0.08
**IA**	0.89 ± 0.17	0.85 ± 0.1	1.97 ± 0.4	1.39 ± 0.25	1.37 ± 0.19
**SA**	1.86 ± 0.34	1.08 ± 0.08	1.18 ± 0.26	1.1 ± 0.19	2.61 ± 0.55
τ_{inact} **(ms)**	**17.32 ± 5.02**	**69.29 ± 19.78******	**22.15 ± 5.78**	**62.41 ± 16.36*****	**45.57 ± 16.44****
**RA**	0.9 ± 0.1	1.58 ± 0.23	1.32 ± 0.17	1.6 ± 0.26	1.47 ± 0.14
**IA**	16.92 ± 3.29	15.59 ± 2.84	22.71 ± 2.52	21.91 ± 3.56	16.56 ± 3.26
**SA**	136.9 ± 22.05	235.3 ± 55.26	142.2 ± 33.37	230.8 ± 41.94	257.4 ± 85.34

Sex and number of animals (m, male, f, female) used in the study. Cells and currents analysed in nociceptors for each genotype are shown. Cell size and half peak (HP) of APs were used to classified DRG neurones into nociceptors. Mechanical latency, τ_{act} and τ_{inact} are shown for all genotypes including the values or RA, IA and SA groups. (Kruskal-Wallis test, *P<0.05, **P<0.01,***P<0.001, ****P<0.0001). Values are shown as mean ± s.e.m.

Supplementary Table 6. Biophysical properties of indentation-gated currents on nociceptors from *Piezo2*$^{R2756H/R2756H}$ and *Piezo2*$^{R2756K/R2756K}$ mice

	Piezo2$^{+/+}$	***Piezo2***$^{R2756H/R2756H}$	***Piezo2***$^{R2756K/R2756K}$
**Animals**	4	3	3
**Cells**	10	19	18
**Cell size (µm)**	28.5 ± 1.2	26.4 ± 0.7	27.2 ± 0.6
**HP (ms)**	1.5 ± 0.2	1.6 ± 0.2	1.6 ± 0.2
**Em_{rep} (mV)**	-56.1 ± 2.6	-51.2 ± 2.3	-55.0 ± 1.8
**Mechanical threshold (µm)**	8.1 ± 0.6	7.6 ± 0.4	**6.0 ± 0.4***
**I_{max} (pA)**	86.2 ± 23.8	159.4 ± 73.1	285.3 ± 96.5
**τ_{act} (ms)**	5.4 ± 1.5	4.6 ± 0.6	4.4 ± 0.4
**τ_{inact} (ms)**	27.0 ± 8.2	76.4 ± 35.1	71.2 ± 37.1
***RA* τ_{inact} (ms)**	1.4 ± 0.2	**5.2 ± 1.0***	**5.1 ± 0.8***

Number of animals and responsive cells analysed in the study. Cell size and half peak (HP) of APs were used to classify DRG neurones into nociceptors. (Mechanical threshold: Kruskal-Wallis test, *P=0.02; τ_{inact}: Kruskal-Wallis test, *P<0.05). Values are shown as mean ± s.e.m.

Supplementary Table 7. Gating measures from *Piezo2*$^{+/+}$ and *Piezo2 knock in* mice

	***Piezo2*$^{+/+}$**	***Piezo2*$^{R2756H/R2756H}$**	***Piezo2*$^{R2756K/R2756K}$**
**Speed (cm/s)**	5.8 ± 0.8	5.2 ± 0.3	7.2 ± 0.9
**Swing speed (cm/s)**	3.2 ± 0.1	2.6 ± 0.1	3.6 ± 0.4
**Body linearity index**	1.5 ± 0.09	1.2 ± 0.05*	1.5 ± 0.1
**Step length (anterior legs, mm)**	29.2 ± 1.6	24.5 ± 1.0	26.4 ± 1.9
**Step length (posterior legs, mm)**	27.7 ± 1.8	23.4 ± 1.2	26.4 ± 2.0

Mouse walk behaviour assay showed similar motor coordination in *Piezo2*$^{+/+}$ and *Piezo2 knock in* mice. The speed was calculated from the velocity of the animal to cross from one side of the walkway to the other. Swing speed represents the movement of the paw from the standing place to the next position. The body linearity index indicates the ability of the animal to walk in a straight line considering the position of the body of stance (Kruskal-Wallis test, *P=0.02). Step length shows the distance covered from the movement of the paw from the standing place to the next position. (*n*: *Piezo2*$^{+/+}$, 7; *Piezo2*$^{R2756H/R2756H}$, 8; *Piezo2*$^{R2756K/R2756K}$, 5)

Supplementary Methods

Cultured cell lines

N2a$^{Piezo1-/-}$ cells were used for characterizing the biophysical properties of the mutants of mPiezo2 and the chimeric channel mP1/mP2. Recently we showed that these cells lack stretch- and deflection-gated currents[1,2]. Cells were cultured in 45% DMEM-Glutamax (gibco, ThermoFisher SCIENTIFIC), 45% Opti-MEM (gibco, ThermoFisher SCIENTIFIC), 10% fetal calf serum (PAN Biotech GMBH) and 1% penicillin and streptomycin (Sigma-Aldrich) media.

N2a$^{Piezo1-/-}$ cells were transiently transfected using FuGeneHD (Promega, Madison). A mix of 100 μL of Opti-MEM, 3 μL of FuGeneHD and 1 μg of DNA was incubated for 10 min at room temperature. The mix was added to Na2$^{Piezo1-/-}$ cells cultured in 30 mm x 15 mm Petri dishes and 900 μL of media containing 50% DMEM-Glutamax and 50% Opti-MEM was added for an overnight transfection. Electrophysiological recordings were made 18-24 h post-transfection. At least four transfections were made for each set of experiments.

Electrophysiology; Mechanical Stimulation

For pillar arrays experiments, a single pilus was deflected using a heat-polished borosilicate glass pipette (mechanical stimulator) driven by a MM3A micromanipulator (Kleindiek Nanotechnik, Germany) as described in Poole et al., 2014[3]. Only cells on the top of pili were stimulated. Mechanically-gated currents were classified according to their inactivation kinetics as previously described: the rapidly adapting (RA, τ_{inact} <5 ms), intermediate adapting (IA, τ_{inact} 5-50 ms) and slowly adapting currents (SA, τ_{inact} > 50ms)[3]. Pillar deflection stimuli were applied in the range of 1-1000 nm, larger deflections were discarded. For quantification and comparison analysis, the data was binned by the magnitude of the stimuli (1-10, 11-50, 51-100, 101-250, 251-500, 501-1000 nm) and calculated the mean of the current amplitudes within each bin for every cell. Bright field images (Zeiss 200 inverted microscope) were collected using a 40X objective and a CollSnapEZ camera (Photometrics, Tucson, AZ) before and after the pillar stimuli to calculate the pillar deflection. The pillar movement was calculated comparing the light intensity of the center of each pilus before and after the stimuli with a 2D-Gaussian fit (Igor Software, WaveMetrics, USA).

High speed pressure clamp (HSPC, Ala Scientific) experiments were carried out in excised outside-out patched pulled from transiently transfected N2a$^{Piezo1-/-}$ cells. Recording pipettes had a final resistance of 6-8 MΩ. Positive pressure pulses were applied through the recording

electrode. Pressure steps protocol consisted in ranging stimuli from 10 to 170 mmHg, in 20 mmHg steps while holding the patch potential at -60 mV. Tail currents protocol consisted of applying depolarized pre-pulses ranging from -60 to 140 mV followed by a repolarizing voltage step to -60 mV in the continuous presence of pressure. Recording solutions consisted in symmetrical ionic conditions containing (in mM): 140 NaCl, 10 HEPES, 5 EGTA adjusted to pH 7.4 with NaOH[1]. Currents were recorded at 10 KHz and filtered at 3 KHz using an EPC-10 USB amplifier (HEKA, Elektornik GmbH, Germany) and Patchmaster software.

For indentation experiments, N2a$^{Piezo1-/-}$ cells and isolated DRG neurones were clamped at -60 mV in whole-cell configuration. Mechanical stimulation was performed on the soma using a heat-polished blunt borosilicate glass pipette (tip size 3-4 μm). A series of indentation stimuli were applied to all cell bodies ranging from ~0.6 up to 10.5 μm, each pulse lasting 500 ms with 7 s intervals. Only cells that did not detach and resisted more that 6 stimuli (pulse of ~6.5 μm) were considered into the analysis. Mechanically activated currents were classified according to their inactivation kinetics: rapidly adapting (RA, τ_{inact} <10 ms), intermediate adapting (IA, τ_{inact} 10-50 ms) and slowly adapting currents (SA, τ_{inact} > 50ms) as previously described[4,5].

Ex vivo skin nerve recordings

In all preparations, the skin and nerve were dissected free and transferred to the recording chamber where muscle, bone and tendon tissues were removed from the skin to improve recording quality. The recording chamber was perfused with a 32°C synthetic interstitial fluid (SIF buffer): 123 mM NaCl, 3.5 mM KCl, 0.7mM MgSO4, 1.7 mM NaH2PO4, 2.0 mM CaCl2, 9.5 mM sodium gluconate, 5.5 mM glucose, 7.5 mM sucrose and 10 mM HEPES (pH7.4). The skin was pinned out and stretched, such that the outside of the skin could be stimulated using stimulator probes. The peripheral nerve was fed through to an adjacent chamber in mineral oil, where fine filaments were teased from the nerve and placed on a silver wire recording electrode.

The receptive fields of individual mechanoreceptors were identified by mechanically probing the surface of the skin with a blunt glass rod or blunt forceps. Analog output from a Neurolog amplifier were filtered and digitized using the Powerlab 4/30 system and Labchart 7.1 software (ADinstruments). Spike-histogram extension for Labchart 7.1 was used to sort spikes of individual units. Electrical stimuli (1Hz, square pulses of 50-500 µs) were delivered to single-unit receptive fields to measure conduction velocity and enable classification as C-fibres (velocity <1.2 ms-1), A-δ fibres (1.2-10 ms-1) or A-β fibres (>10 ms-1). Mechanical stimulation of the receptive fields of neurones were performed using a piezo actuator (Physik

Instrumente, P-841.60) and a double-ended Nanomotor (Kleindiek Nanotechnik, MM-NM3108) connected to a force measurement device (Kleindiek Nanotechnik, PL-FMS-LS). Calibrated force measurements were acquired simultaneously using the Powerlab system and Labchart software during the experiment.

As different fibre types have different stimulus tuning properties, different mechanical stimuli protocols were used based on the unit type. Low threshold Aβ-fibres were stimulated with a 25 Hz vibration stimulus with increasing amplitude over 6 steps (from ~6-44 mN; 20 cycles per step), and a dynamic stimulus sequence with four ramp and hold waveform with varying probe deflection velocity (3 s duration; 0.075 mms-1, 0.15 mms-1, 0.45 mms-1 and 1.5 mms-1; average amplitude 100 mN). Aβ-fibre slowly-adapting mechanoreceptors (SAMs) and rapidly-adapting mechanoreceptors (RAMs) were classified by the presence or absence of firing during the static phase of a ramp and hold stimulus, respectively as previously described[6,7]. Single-units were additionally stimulated with a series of five static mechanical stimuli with ramp and hold waveforms of increasing amplitude (3 s duration; ranging from ~10-160 mN). High threshold Aδ- and C-fibres were also stimulated using the five ramp and hold stimuli with increasing amplitudes. Spontaneous activity in C-fibres was analysed after every mechanical stimuli.

Behavioural testing

Brush and von Frey experiments

For all behavioural testing the animals were tested in a dedicated behavioural testing room during the light phase. Adult males and females were included in the experiments. The experimenter was always blind to the genotype of the tested animals.

Mice were placed in plastic cages with a metal grid floor bottom that allowed access to hindpaw stimulation. Animals were habituated for two-consecutives days for 20 min before testing. Before starting the behavioural test, animals were placed in the cages for at least 20 min (accommodation) and experimentation started when animals stopped cage exploration. Each animal was tested at least twice on different days and average values from those measures were plotted for each animal.

Responses to gentle touch was measured by stroking the surface of the hindpaw. Animals were stimulated five times with at least 2 min between each stimulation. The percentage was calculated according to the number of withdrawals out of the five stimulations.

Calibrated von Frey (Semmes-Weinstein) filaments (Aesthesio®, USA) were used to test the 50% paw withdrawal threshold (50% PWT) in mice. The "up-down method" was used to

calculate the 50% PWT[8–10]. Mid-plantar right and left hindpaw were stimulated for approximately 1-3 s with von Frey filaments in the range of 0.008, 0.02, 0.04, 0.07, 0.16, 0.4, 0.6, 1.0, 1.4 and 2 g. Von Frey filaments were presented perpendicular to hind paws at intervals of 3-5 min. A positive response was considered when the paw was withdrawn.

Hargreaves assay

To test thermal pain sensation, mice were habituated on a Plexiglas apparatus for 30 min until minimal movement was observed. The hind paws of the animals were stimulated from below using an infrared light source (IR 30; Ugo Basile, 30570). Thermal paw withdrawal latencies were recorded. Three different measures were recorded per animal with a time interval between 3-5 min.

Beam Test

Mice were first trained to cross a 100 cm long, 25 mm circular wide beam. After the training, animals were tested on the elevated 100 cm long (labeled every 10 cm), 10 mm circular beam. At least three trials were recorded using a monochromatic high-speed camera (FLIR, RoHS 2.3 MP Mono Grasshopper; 30 frames/second) and a Canon EF-S, 18-55 mm lens. A mirror angled 45° below the beam was included in the recordings (image/reflection). The total of slips in the three runs were calculated by analyzing the videos frame by frame using Fiji22. Total of slips were normalized according to the total distance crossed by the animals with a minimum distance of 80 cm/trial.

Horizontal Ladder Test

Animals were placed on a 40 cm horizontal ladder (rung distance: 1 cm). Similar to the beam test, at least three trials were recorded using a monochromatic high-speed camera (FLIR, RoHS 2.3 MP Mono Grasshopper; 60 frames/second) and a Canon EF-S, 18-55 mm lens. A mirror angled 45° below the ladder was used as image/reflection. The total of foot slipping or dropping down in between the rungs (errors) in the three runs were calculated by analyzing the videos frame by frame using Fiji22. Total of errors were normalized according to the total distance crossed by the animals with a minimum distance of 36 cm/trial.

Digging assay

A 49 × 10 × 12 cm cage with a wire bar lid was filled with Aspen midi 8/20 wood chip bedding (LBS Biotechnology) which was tamped down to a depth of ∼4 cm as previously described[11]. A mouse was put in a cage individually with fresh bedding and the cage was placed inside a Multi Conditioning System (TSE systems) where mice behavior was recorded for 5 min using

a Digital Color CCD Camera CM-S4400C (Cameray). To avoid distractions food and water were not available during the recordings.

Gait Analysis

Gait analysis was carried out using the "mouse walk" method as previously described[12,13]. An 80 cm length acrylic walkway was produced with white-light LED strips attached to its sides. To generate a light and even background (sky), a similar walkway was set with white plastic attached to the top. These two walkways were embedded in a black custom-built rack with a mirror angled 45° below the walkway (image/reflection). A monochromatic high-speed camera (FLIR, RoHS 2.3 MP Mono Grasshopper; 150 frames/s) was used to record the animal's walk. No additional light source was present in the room. Animals were first habituated for 15 min by allowing them to explore freely on the walkway. Then, animals were placed on the side of the walkway and crossings were recorded. Each animal was recorded multiple times to obtain recordings with constant velocity and no stops during the crossing. Videos were converted into single image stacks and analyzed using a MATLAB script provided by Cesar Mendes (iNOVA4Health, NOVAMedical School, Faculdade de Ciências Médicas, Universidade Nova de Lisboa, Lisboa, Portugal).

References

1. Moroni M, Servin-vences MR, Fleischer R, Sánchez-Carranza O, Lewin GR. Voltage gating of mechanosensitive PIEZO channels. *Nature Communications*. 2018;(2018):1-15. doi:10.1038/s41467-018-03502-7

2. Patkunarajah A, Stear JH, Moroni M, et al. TMEM87a/Elkin1, a component of a novel mechanoelectrical transduction pathway, modulates melanoma adhesion and migration. *eLife*. 2020;9:1-25. doi:10.7554/eLife.53308

3. Poole K, Herget R, Lapatsina L, Ngo HD, Lewin GR. Turning Piezo ion channels to detect molecular-scale movements revelant for fine touch. *Nature Communications*. 2014;5. doi:10.1038/ncomms4520

4. Wetzel C, Hu J, Riethmacher D, et al. A stomatin-domain protein essential for touch sensation in the mouse. *Nature*. 2007;445(January):206-209. doi:10.1038/nature05394

5. Hu J, Lewin GR. Mechanosensitive currents in the neurites of cultured mouse sensory neurones. *J Physiol*. 2006;577(Pt 3):815-828. doi:10.1113/jphysiol.2006.117648

6. Walcher J, Ojeda-Alonso J, Haseleu J, et al. Specialized mechanoreceptor systems in rodent glabrous skin. *J Physiol*. 2018;596(20):4995-5016. doi:10.1113/JP276608

7. Milenkovic N, Wetzel C, Moshourab R, Lewin GR. Speed and Temperature Dependences of Mechanotransduction in Afferent Fibers Recorded From the Mouse Saphenous Nerve. *Journal of Neurophysiology*. 2008;100(5):2771-2783. doi:10.1152/jn.90799.2008

8. Chaplan SR, Bach FW, Pogrel JW, Chung JM, Yaksh TL. Quantitative assessment of tactile allodynia in the rat paw. *J Neurosci Methods*. 1994;53(1):55-63. doi:10.1016/0165-0270(94)90144-9

9. Dixon WJ. Efficient analysis of experimental observations. *Annu Rev Pharmacol Toxicol*. 1980;20:441-462. doi:10.1146/annurev.pa.20.040180.002301

10. Christensen SL, Hansen RB, Storm MA, et al. Von Frey testing revisited: Provision of an online algorithm for improved accuracy of 50% thresholds. *Eur J Pain*. 2020;24(4):783-790. doi:10.1002/ejp.1528

11. Chakrabarti S, Pattison LA, Singhal K, Hockley JRF, Callejo G, Smith ESJ. Acute inflammation sensitizes knee-innervating sensory neurons and decreases mouse digging behavior in a TRPV1-dependent manner. *Neuropharmacology*. 2018;143:49-62. doi:10.1016/j.neuropharm.2018.09.014

12. Mendes CS, Bartos I, Márka Z, Akay T, Márka S, Mann RS. Quantification of gait parameters in freely walking rodents. *BMC Biol*. 2015;13:50. doi:10.1186/s12915-015-0154-0

13. Ojeda-Alonso J, Bégay V, Garcia-Contreras JA, Campos-Pérez AF, Purfürst B, Lewin GR. Lack of evidence for participation of TMEM150C in sensory mechanotransduction. *J Gen Physiol*. 2022;154(12):e202213098. doi:10.1085/jgp.202213098
